# Combinatorial metabolic engineering of *Pseudomonas putida* KT2440 for efficient mineralization of 1,2,3-trichloropropane

**DOI:** 10.1038/s41598-017-07435-x

**Published:** 2017-08-01

**Authors:** Ting Gong, Xiaoqing Xu, You Che, Ruihua Liu, Weixia Gao, Fengjie Zhao, Huilei Yu, Jingnan Liang, Ping Xu, Cunjiang Song, Chao Yang

**Affiliations:** 1 Key Laboratory of Molecular Microbiology and Technology for Ministry of Education, Nankai University, Tianjin, 300071 China; 2 State Key Laboratory of Medicinal Chemical Biology, Nankai University, Tianjin, 300071 China; 3 State Key Laboratory of Bioreactor Engineering, East China University of Science and Technology, Shanghai, 200237 China; 4 Core Facility of Equipment, Institute of Microbiology, Chinese Academy of Sciences, Beijing, 100101 China; 5 State Key Laboratory of Microbial Metabolism, and School of Life Sciences & Biotechnology, Shanghai Jiao Tong University, Shanghai, 200240 China

## Abstract

An industrial waste, 1,2,3-trichloropropane (TCP), is toxic and extremely recalcitrant to biodegradation. To date, no natural TCP degraders able to mineralize TCP aerobically have been isolated. In this work, we engineered a biosafety *Pseudomonas putida* strain KT2440 for aerobic mineralization of TCP by implantation of a synthetic biodegradation pathway into the chromosome and further improved TCP mineralization using combinatorial engineering strategies. Initially, a synthetic pathway composed of haloalkane dehalogenase, haloalcohol dehalogenase and epoxide hydrolase was functionally assembled for the conversion of TCP into glycerol in *P. putida* KT2440. Then, the growth lag-phase of using glycerol as a growth precursor was eliminated by deleting the *glpR* gene, significantly enhancing the flux of carbon through the pathway. Subsequently, we improved the oxygen sequestering capacity of this strain through the heterologous expression of *Vitreoscilla* hemoglobin, which makes this strain able to mineralize TCP under oxygen-limited conditions. Lastly, we further improved intracellular energy charge (ATP/ADP ratio) and reducing power (NADPH/NADP^+^ ratio) by deleting flagella-related genes in the genome of *P. putida* KT2440. The resulting strain (named KTU-TGVF) could efficiently utilize TCP as the sole source of carbon for growth. Degradation studies in a bioreactor highlight the value of this engineered strain for TCP bioremediation.

## Introduction

Synthetic biology has become a powerful tool to construct complete heterologous metabolic pathways in a host cell by functional assembly of various enzymes from different organisms^1^. Currently, most studies have focused on implantation of diverse synthetic pathways into microbial cells to synthesize high value-added products^2^. Although synthetic biology tools are not widely applied for engineering the organics catabolic pathways in microorganisms so far, they have enormous potential for rational tuning of pathways for efficient degradation of environmental pollutants.

A toxic persistent pollutant, 1,2,3-trichloropropane (TCP), has been recognized as an emerging contaminant in groundwater. The global yield of TCP can reach about 50,000 tons annually, and TCP is widely used as a solvent and as building block for the synthesis of other chemicals^3^. TCP is frequently detected in groundwater due to improper waste disposal, its moderate water solubility, and its recalcitrance to biodegradation^4^. TCP contamination of groundwater poses a serious threat to drinking water sources and human health. Therefore, there is an urgent need to develop efficient technologies for the remediation of TCP-contaminated sites.

Disposal of TCP by physical or chemical methods is expensive. Bioremediation, which is a simple, safe and cost-effective technique to fight pollution, utilizes and manipulates the biodegradation abilities of living organisms to transform toxic organic pollutants into harmless products^5^. To date, there are no reports on the aerobic degradation of TCP by natural microorganisms. Metabolic engineering has opened up new avenues for the evolution of efficient degradation pathways^6^, which allows the construction of recombinant microorganisms with the capability to degrade TCP under aerobic conditions.

Haloalkane dehalogenases (DhaA) catalyze the dehalogenation of TCP to the (*R*) and (*S*) enantiomers of 2,3-dichloropropane-1-ol (DCP)^7^. To improve the catalytic efficiency and enantioselectivity, two DhaA mutants^8, 9^, DhaA31 and DhaA90R, were generated by directed evolution. Furthermore, haloalcohol dehalogenases (HheC) and epoxide hydrolases (EchA) are responsible for the conversion of DCP to glycerol (GLY)^10, 11^. Recently, a synthetic biodegradation pathway capable of aerobic biotransformation of TCP into harmless GLY was assembled by the heterologous expression of DhaA, HheC and EchA in *Escherichia coli* ^12, 13^. However, expression of the synthetic pathway is dependent on IPTG induction, which is not suitable for practical application in the large-scale degradation of TCP. Moreover, the plasmid-based expression systems tend to lose the introduced heterologous genes in the absence of selective pressure and the diffusion of antibiotic resistance markers on plasmids poses potential risks to the environment. More importantly, lab-born *E. coli* strains are not regarded as good candidates for *in situ* bioremediation due to their strict nutrient demand, poor adaptability and weak competitiveness. In another study, a *dhaA31* gene encoding DhaA31 was integrated into the chromosome of the DCP-degrading bacterium *Pseudomonas putida* MC4 using a transposon delivery system^4^. However, the engineered strain needs to be intensively researched with regard to its biosafety prior to its application for *in situ* bioremediation due to legislative barriers on the field release of genetically engineered microorganisms.

The GRAS (generally recognized as safe) strain *P. putida* KT2440 is considered as a potential agent for environmental bioremediation of industrial waste. The genome of *P. putida* KT2440 has been sequenced and multiple tools for genome editing have been devised and implemented^14–18^, which have laid a foundation for the metabolic engineering of *P. putida* KT2440. Recently, *P. putida* KT2440 has been highlighted as a robust metabolic chassis for catabolic pathway assembly^19^.

**Figure 1:**
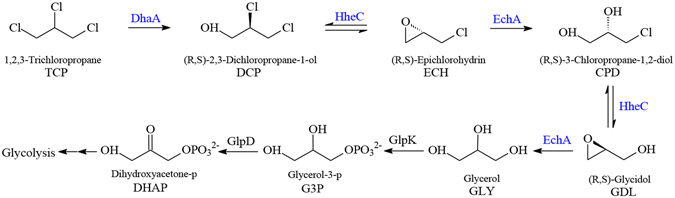
Construction of a synthetic pathway for aerobic mineralization of TCP in *P. putida* KT2440. Enzyme sources: DhaA from *Rhodococcus rhodochrous* NCIMB 13064, HheC from *Agrobacterium radiobacter* AD1, and EchA from *Agrobacterium radiobacter* AD1.

In this work, we engineered *P. putida* KT2440 for the efficient degradation of TCP using combinatorial engineering strategies by carrying out the following four tasks: (1) constructed a synthetic pathway for the conversion of TCP into GLY (Fig. 1), in particular, prochiral TCP was converted predominantly into (*R*)-DCP (ee 90%) by the enantioselective DhaA90R and the accumulation of (*S*)-DCP in the pathway caused by the poor activity of HheC toward (*S*)-DCP was avoided; (2) deleted the *glpR* gene to eliminate the growth lag-phase of using GLY as a carbon source; (3) enhanced aerobic metabolism by promoting oxygen delivery through the heterologous expression of *Vitreoscilla* hemoglobin (VHb); (4) increased intracellular energy charge (ATP/ADP ratio) and reducing power (NADPH/NADP^+^ ratio) by deleting the flagellar operon (a ~ 70 kb DNA segment of the genome). Through the engineering of TCP biodegradation pathway, the enhancement of oxygen transport, and the modulation of energy status and reducing power availability, the resulting strain could mineralize TCP aerobically and utilize TCP as the sole source of carbon for growth.

## Results and Discussion

### Construction of a synthetic TCP mineralization pathway in *P. putida* KT2440

Currently, synthetic biology approaches can be applied for implantation of the synthetic biodegradation pathways into an ideal host strain for bioremediation^20^. In this work, a synthetic pathway for aerobic mineralization of TCP comprising DhaA from *Rhodococcus rhodochrous* NCIMB 13064, HheC from *Agrobacterium radiobacter* AD1, and EchA from *Agrobacterium radiobacter* AD1 was assembled in *P. putida* KT2440 (Fig. 1). All these genes (*dhaA90R*, *hheC* and *echA*) were integrated into the chromosome of *P. putida* KT2440 using a previously developed genome editing method with *upp* as a counter-selectable marker^16^. To improve the efficiency of the synthetic biodegradation pathway, the *vgb* gene was inserted into the *P. putida* chromosome, and both the *glpR* gene and the flagellar operon were knocked out. The resulting mutant strain was designated as *P. putida* KTU-TGVF. The successful construction of strain KTU-TGVF was verified by PCR and DNA sequencing. The correct amplicons were obtained by PCR with chromosomal DNA of strain KTU-TGVF as the template (Fig. S1). The nucleotide sequences of the inserted DNA fragments on chromosome were in accordance with those of four synthetic gene cassettes (Fig. S2).

The poor activity of DhaA toward TCP and cellular toxicity of TCP and intermediates represent primary bottlenecks of the TCP mineralization pathway, limiting the flux of carbon through the pathway^3, 12^. To overcome the bottlenecks of aerobic TCP mineralization, previous studies focused on engineering of DhaA, the first enzyme of the TCP mineralization pathway. Two DhaA mutants, DhaA31 and DhaA90R, were obtained using computer-assisted directed evolution. Mutant DhaA31^8^ showed 26-fold higher catalytic efficiency toward TCP than the wild-type enzyme (DhaAwt: *k*_cat_ = 0.08 s^−1^, *K*_m_ = 2.2 mM, *k*_cat_/*K*_m_ = 36 M^−1^ s^−1^; DhaA31: *k*_cat_ = 1.26 s^−1^, *K*_m_ = 1.2 mM, *k*_cat_/*K*_m_ = 1050 M^−1^ s^−1^), while the (*S*)-DCP was accumulated in the pathway because of equimolar production of (*R*)-DCP and (*S*)-DCP by the non-enantioselective DhaA31 and the poor activity of HheC toward (*S*)-DCP. Mutant DhaA90R^9^ possessed similar catalytic efficiency toward TCP as the wild-type enzyme (DhaA90R: *k*_cat_ = 0.16 s^−1^, *K*_m_ = 6.5 mM, *k*_cat_/*K*_m_ = 25 M^−1^ s^−1^), while DhaA90R converted prochiral TCP predominantly into (*R*)-DCP (ee 90%), which is the preferred substrate for the enantioselective HheC. In a previous study, the synthetic pathway with DhaA90R reconstructed in *E. coli* BL21 (DE3) failed to provide sufficient carbon flux to support bacterial growth in minimal medium with TCP^12^. In another study, a novel TCP biodegradation pathway including a chromosome-borne *dhaA31* gene and an intrinsic nonselective DCP degradation pathway was reconstructed in a natural DCP-degrading bacterium *P. putida* MC4. Because of cellular toxicity of TCP and intermediates, the engineered *P. putida* MC4 showed a prolonged growth lag-phase when cultured in minimal medium with TCP^4^. To construct a practically applicable TCP-degrading bacterium, in this study, a robust environmental bacterium *P. putida* KT2440, which is resistant to toxic organic solvents, was chosen as a host strain for reconstructing the TCP mineralization pathway. To avoid the accumulation of (*S*)-DCP in the pathway, in this study, the enantioselective DhaA90R was selected for the dehalogenation of TCP to DCP. As discussed later in detail, the insufficient carbon flux through the pathway caused by the less active DhaA90R was further improved by eliminating the growth lag-phase of using GLY as a carbon source, enhancing aerobic metabolism, and increasing intracellular energy charge and reducing power.

### Heterologous expression of the synthetic TCP mineralization pathway in *P. putida* KT2440

To achieve the optimal expression levels, the optimized gene expression regulatory elements^21^, including a strong constitutive promoter, two Shine-Dalgarno sequences, and a terminator, were designed for controlling the transcription and translation of exogenous genes (Fig. S3). In order to verify whether these exogenous genes (*dhaA90R*, *hheC*, *echA* and *vgb*) are transcribed to mRNA in *P. putida* KTU-TGVF, RT-PCR assays were performed. As expected, these target products were obtained by PCR with cDNA or genomic DNA as template. In contrast, no PCR products were obtained with mRNA or ddH_2_O as template (Fig. S4). These results indicated that transcription of these exogenous genes had occurred in *P. putida* KTU-TGVF.

Production of DhaA, HheC and EchA in KTU-TGVF cells was demonstrated by Western blot analysis. Immunoreactive bands, which matched well with the theoretical molecular weights of DhaA (34 kDa), HheC (29 kDa) and EchA (35 kDa), were detected in whole-cell lysates using antibodies against DhaA, HheC and EchA (Fig. S5).

### Function validation for the synthetic TCP mineralization pathway

**Figure 2:**
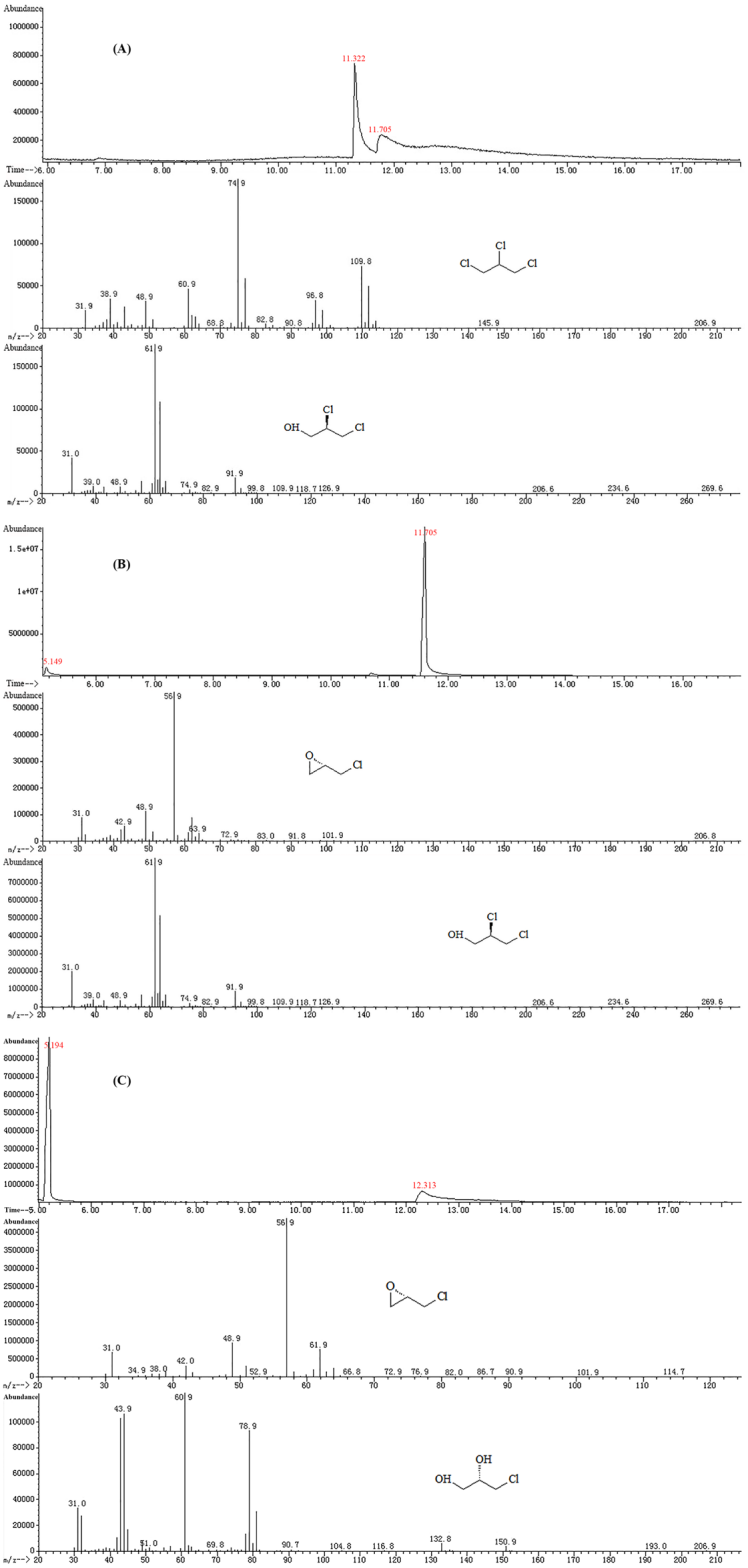
(**A**) GC-MS analysis of transformation of TCP by resting cells of *P. putida* KTU-T1. Two chromatographic peaks representing TCP and DCP had a RT of 11.3 min and 11.7 min, respectively. (**B**) GC-MS analysis of transformation of DCP by resting cells of *P. putida* KTU-T2. Two chromatographic peaks corresponding to ECH and DCP appeared at a RT of 5.1 min and 11.7 min, respectively. (**C**) GC-MS analysis of transformation of ECH by resting cells of *P. putida* KTU-T3. Two chromatographic peaks representing ECH and CPD had a RT of 5.1 min and 12.3 min, respectively.

The functionality of DhaA, HheC and EchA in KTU-TGVF cells was validated by GC-MS analyses of resting-cell transformation products. Resting cells of KTU-T1 containing *dhaA90R* produced a new chromatographic peak with a retention time (RT) of 11.7 min when incubated with TCP, and this peak was identified as DCP by comparing its MS pattern with that of DCP standard (Fig. 2A). Transformation of DCP by resting cells of KTU-T2 containing *hheC* produced a new chromatographic peak with a RT of 5.1 min, which had the same RT and MS pattern as ECH standard (Fig. 2B). After the transformation of ECH by resting cells of KTU-T3 containing *echA*, a new chromatographic peak appeared at a RT of 12.3 min, the RT and MS pattern of this peak were the same as those of CPD standard (Fig. 2C). Therefore, KTU-T1, KTU-T2 and KTU-T3 cells expressed functional DhaA, HheC and EchA, respectively.

**Figure 3:**
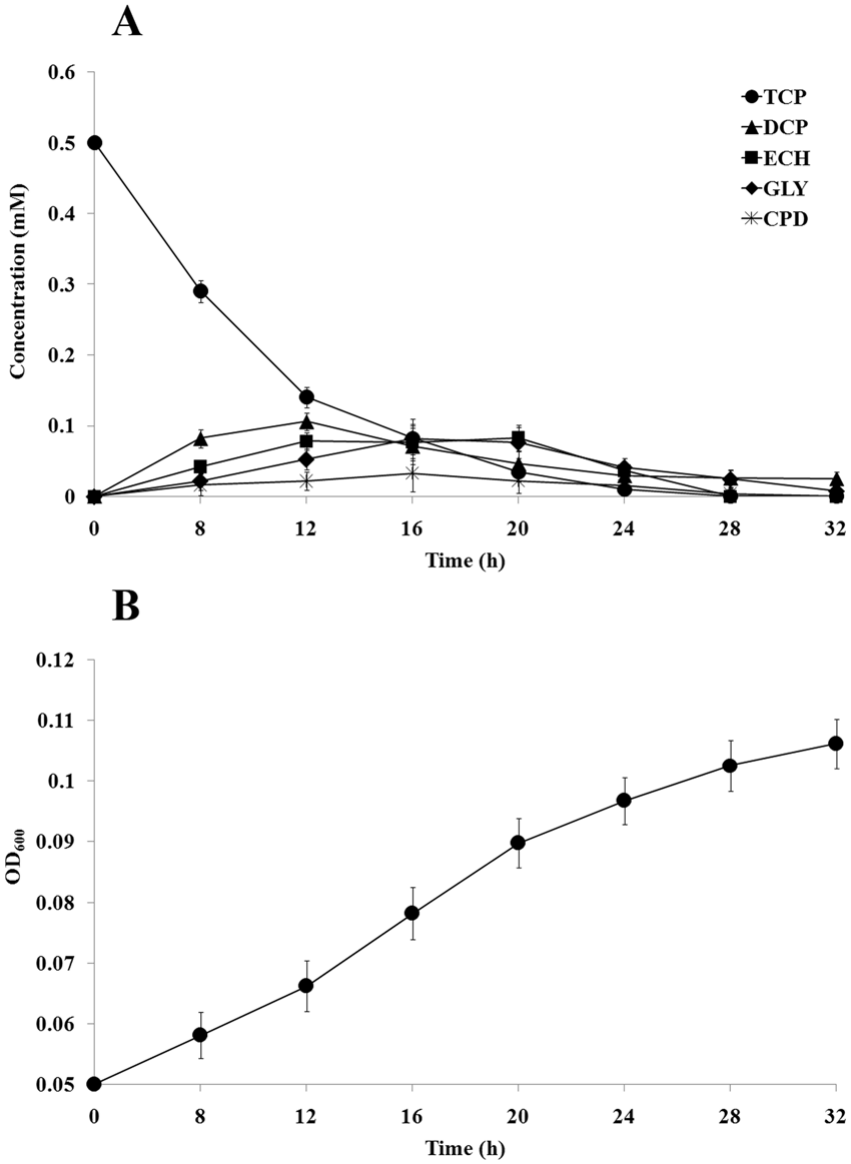
(**A**) Aerobic mineralization of TCP by *P. putida* KTU-TGVF. *P. putida* KTU-TGVF was incubated in M9 minimal medium supplemented with 0.5 mM TCP in a shaking incubator at 200 rpm and 30 °C. The initial inoculum density was OD_600_ = 0.05. TCP, DCP, ECH and CPD were quantified by GC-MS. GLY was quantified by colorimetric analysis. (**B**) Growth curve of *P. putida* KTU-TGVF. The OD_600_ was measured to estimate cell growth. Bars represent the mean values ± standard deviation of triplicate measurements from three independent experiments.

TCP degradation experiment was performed with KTU-TGVF in M9 minimal medium supplemented with 0.5 mM TCP. As shown in Fig. 3A, 0.5 mM TCP was almost completely converted to GLY and the vast majority of GLY were further metabolized within 32 h, as revealed by quantification of DCP, ECH, CPD and GLY throughout the experiment. Accompanying with TCP degradation, cell growth was observed (Fig. 3B), which indicated that this strain could utilize TCP as the sole source of carbon for growth. In contrast, the concentration of TCP did not change and no growth was observed when inoculated with KTU. These results indicated that TCP could be converted to GLY by the heterologous pathway assembled in *P. putida* KT2440 (Fig. 1). Furthermore, GLY could be utilized as the carbon source for cell growth via the intrinsic pathway of KT2440. Taken together, we concluded that the pathway for aerobic mineralization of TCP was functionally assembled in *P. putida* KT2440.

### Elimination of the growth lag-phase of using GLY as a carbon source

GlpR functions as a negative regulator for controlling the utilization of GLY as a growth precursor in *P. putida* KT2440 and the regulatory mechanisms have been elucidated^22^. Deletion of *glpR* may eliminate the growth lag-phase of using GLY as a carbon source. In this work, the strain KTU-TGVF (Δ*glpR*) could grow aerobically without the lag-phase in minimal medium with TCP as the sole carbon source. In contrast, no growth was observed with the strain KTU-T123 (*glpR* ^+^) when using TCP as the sole carbon source. These results suggest that the efficient utilization of GLY may support bacterial growth, alleviate cellular toxicity of TCP and intermediate metabolites and improve the flux of carbon through the TCP mineralization pathway.

### VHb enhances TCP mineralization under oxygen-limited conditions

CO-difference spectrum assays were used to detect VHb activity. The cell extracts from the strain KTU-TGVF showed a characteristic absorption peak at 420 nm when fed with CO, while the peak was not observed with the cell extracts from the strain KTU fed with CO (Fig. S6). These results indicated that the strain KTU-TGVF expressed functional VHb.

VHb delivers the O_2_ to the respiratory chain, enhancing ATP generation and NADH consumption at low O_2_ concentrations. This process could improve the efficiency of bacterial aerobic respiration, increase the carbon flux through the tricarboxylic acid cycle and improve the aerobic growth of bacteria. Heterologous expression of VHb in a variety of hosts has been shown to improve cell growth, protein synthesis, metabolite productivity, and bioremediation under oxygen-limited conditions^23, 24^. In a previous study, integrating *vgb* into the chromosomes of *P. aeruginosa* and *Burkholderia* sp. strain DNT could improve growth and degradation of 2,4-dinitrotoluene or benzoic acid under hypoxic conditions^25^. In another study, *P. putida* KT2440 was engineered for the anoxic biodegradation of 1,3-dichloroprop-1-ene by introducing various genes retrieved from facultative anaerobe and aerotolerant bacteria^26^.

**Figure 4:**
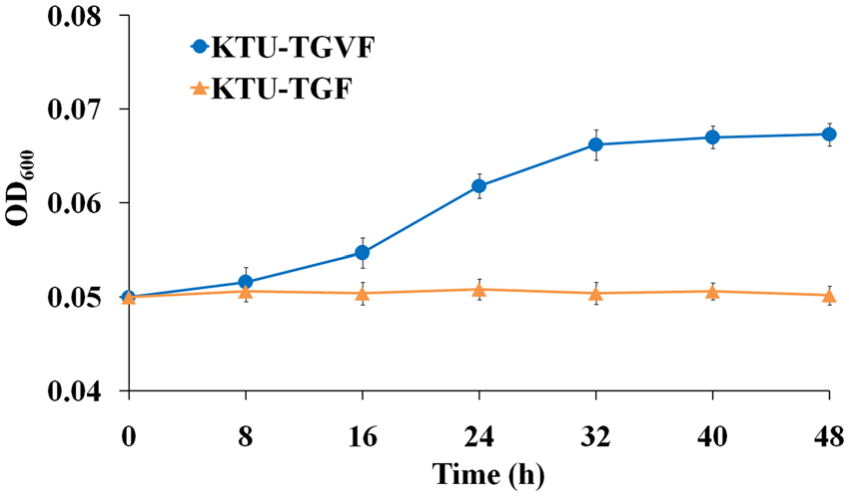
Growth curves of *P. putida* KTU-TGF and KTU-TGVF under oxygen-limited conditions. *P. putida* strains were incubated in 25 ml glass vials with a screw cap mininert valve containing 20 ml of M9 minimal medium supplemented with 0.2 mM TCP in a shaking incubator at 80 rpm and 30 °C. Cell growth was estimated by measuring the OD_600_ of the culture broth. Bars represent the mean values ± standard deviation of triplicate measurements from three independent experiments.

Sufficient oxygen is the key factor for complete oxidative mineralization of TCP. The success of aerobic mineralization of TCP in oxygen-restricted environments will largely depend on the oxygen sequestering capability of TCP-degrading bacteria. In this work, significant growth was observed for the strain KTU-TGVF expressing VHb under oxygen-limited conditions, while no growth was observed for the strain KTU-TGF without VHb expression under oxygen-limited conditions (Fig. 4). These results suggest that VHb may improve the ability of *P. putida* KT2440 to compete for limited oxygen in hypoxic environments, thus making this bacterium more competitive in actual environments such as a packed-bed bioreactor. This study not only underscores the value of *P. putida* KT2440 as a versatile biocatalyst for biotransformation under oxygen-limited conditions but also highlights the value of metabolic engineering for expanding the catalytic repertoire of *P. putida* KT2440.

### Increased intracellular ATP and NADPH levels improve aerobic mineralization of TCP

Both the production and the motion of flagella are energy-consuming processes for the cell. In *E. coli*, flagellar production consumes about 2% of the biosynthetic energy expenditure of the cell, while flagellar motion demands about 0.1% of the total energy cost^27^. In this work, a non-flagellated strain KTU-TGVF was generated from the original strain KTU-TGV by deleting the flagellar operon in the genome. Accordingly, the flagellar operon knockout mutant strain showed the complete absence of the flagellum structure when observed using TEM after negative staining. In contrast, the flagella were observed in the wild-type strain KT2440 (Fig. S7). These results indicated that the flagellar synthesis was completely blocked in the mutant strain.

**Figure 5:**
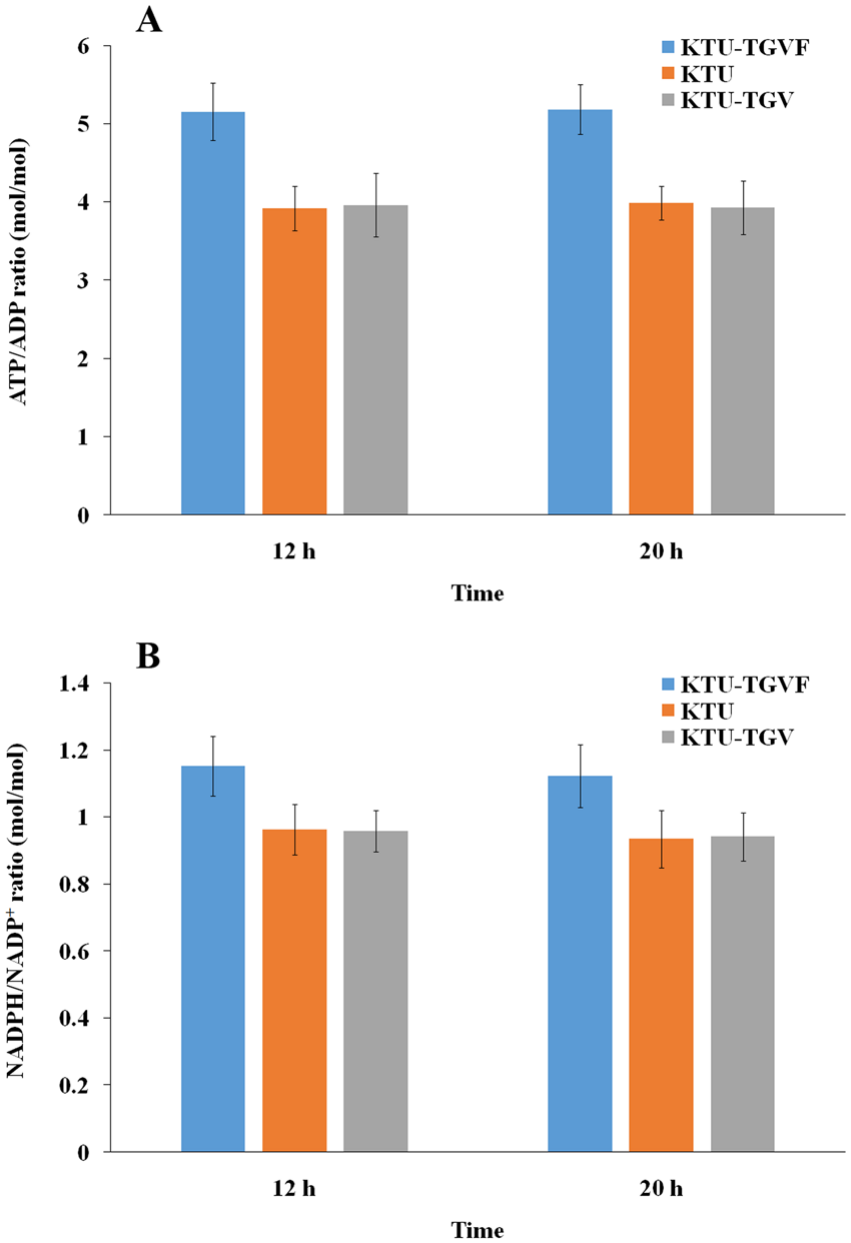
Determination of the ATP/ADP (**A**) and NADPH/NADP^+^ (**B**) molar ratios in *P. putida*. The intracellular levels of ADP, ATP, NADP^+^ and NADPH were determined as described in Materials and methods. Bars represent the mean values ± standard deviation of triplicate measurements from three independent experiments.

It has been reported that the non-flagellated strain KT2440 showed increased intracellular ATP and NADPH levels^28^. In our study, the ATP/ADP ratio in the strain KTU-TGVF was 1.3-fold higher than that in the strain KTU-TGV (Fig. 5A). Since the ATP levels in bacterial cells are tightly regulated, a difference of 30% could significantly influence cellular functions. These results indicated that eliminating the consumption required for flagella synthesis and rotation greatly changed the energy status of the cells.

Except for the energy consumption, a considerable amount of reducing power, mainly in the form of NADPH, is indispensable for basic anabolic processes, such as the biosynthesis of building blocks for biomass^29^. In this work, the strain KTU-TGVF had a 1.2-fold higher NADPH/NADP^+^ ratio than did the strain KTU-TGV (Fig. 5B). Furthermore, the growth kinetics of the strain KTU-TGVF and KTU-TGV were compared. The strain KTU-TGVF showed a higher growth rate in M9 minimal medium containing 0.5 mM TCP than did the strain KTU-TGV. The KTU-TGVF and KTU-TGV cultures reached a maximum OD_600_ of 0.104 and 0.088 at 32 h, respectively (Fig. S8). Obviously, the strain KTU-TGVF could utilize TCP more efficiently as the sole carbon source for growth, which may be a reflection of increased resistance to cellular toxicity of TCP and intermediate metabolites. Increased resistance to toxic substrates may be due in part to enhanced anabolic capability resulting from the increase of ATP and NADPH levels within the cell. Taken together, the efficient mineralization of TCP by the strain KTU-TGVF may be attributed to the efficient utilization of GLY as a growth precursor and the increased intracellular ATP and NADPH levels.

### Treatment of TCP-contaminated water in a lab-scale bioreactor

The flagella-deficient strain KTU-TGVF showed a distinct change in biofilm formation. The quantities of biofilm formed by the strain KTU-TGVF were 4.3- and 9.5-fold higher at 24 and 96 h, respectively, than those formed by the strain KTU-TGV, as judged by the CV staining assay (Fig. S9), when these strains were grown in M9 minimal medium containing 0.4% (w/v) glucose. The observation is in agreement with a previous report^28^, in which the non-flagellated *P. putida* KT2440 cells form more biofilm than the wild-type KT2440 cells. Recently, the biofilm formation capability of *P. putida* KT2440 was improved for boosting biodegradation of haloalkanes by the modulation of the intracellular c-di-GMP level^30^. Biofilm offers protection against a hostile environment and helps bacteria persist within the environment. Therefore, the strain KTU-TGVF has potential for use in a packed-bed bioreactor treating TCP-contaminated water because its high biofilm formation ability is favorable for the immobilization of cells on the support material in wastewater treatment.

**Figure 6:**
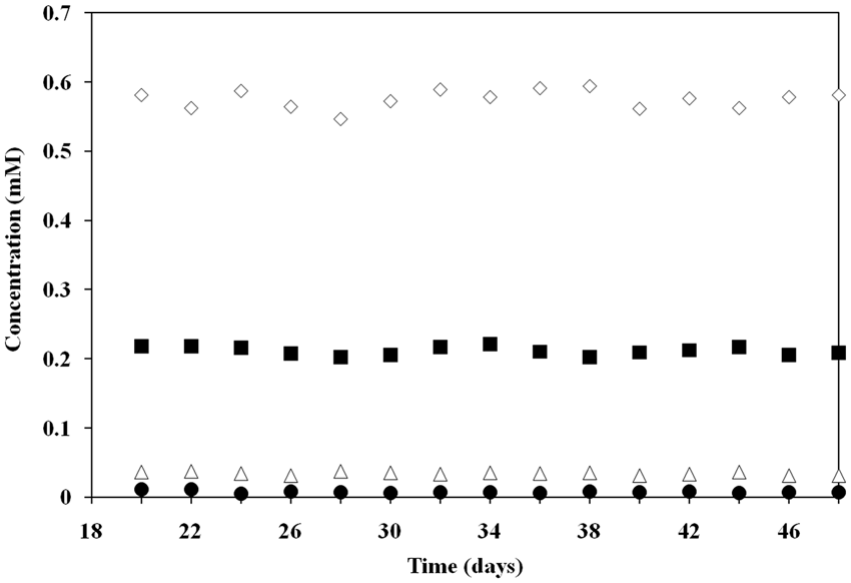
Degradation of TCP by *P. putida* KTU-TGVF in a lab-scale bioreactor. Reactor performance was assessed by continuously monitoring the effluent concentrations of TCP, DCP and chloride during a 30-day operating period. Symbols: ◾, TCP influent; ⚫, TCP effluent; ▵, DCP effluent; ◊, chloride effluent.

To explore the feasibility of using strain KTU-TGVF for TCP bioremediation, we designed a packed-bed bioreactor and tested the removal of TCP from wastewater streams in a consecutive process (Fig. S10). KTU-TGVF cells were grown in M9 minimal medium containing 0.5 mM TCP plus 0.4% glucose to OD_600_ = 0.4 and were subsequently inoculated into the reactor for immobilization on ceramic rings. Previous studies have shown that ceramic rings as the support material are suitable for the immobilization of microbial cells^4, 31^. After inoculation, the reactor was operated under fed-batch conditions for 4 days to promote attachment of the cells to the support material. Within a period of 4 to 18 days, TCP (0.05 to 0.2 mM) was supplied continuously at a rate of 0.1 ml/min. During the operating period, the maintenance of a high hydraulic retention time (HRT, 133 h) may be favorable for cell growth and biofilm formation. HRT is considered as an important operating parameter directly influencing the bioreactor performance^4, 31^. After 18 days, the influent concentration of TCP was maintained at 0.2 mM, and the reactor was continuously operated for another 30 days. The reactor performance was evaluated by continuous monitoring of the effluent concentrations of TCP, DCP and chloride (Fig. 6). Under these operating conditions, the removal efficiencies of TCP were 95 to 97% and quantitative release of chloride (TCP/chloride, molar ratio 1:3) was observed. We detected minute quantities of DCP in the reactor effluent. When inoculated with the strain KTU, the TCP effluent concentration remained unchanged relative to the influent concentration.

Cells from the reactor effluent were spread on LB agar plates and colonies formed were checked for their identity. All 20 colonies tested were identified as *P. putida* KT2440 by the 16 S rRNA gene sequencing. Furthermore, both the four gene insertions (*dhaA90R* ^+^, *hheC* ^+^, *echA* ^+^, *vgb* ^+^) and the two gene deletions (∆*glpR*, ∆flagellar operon) were detected by PCR from all 20 colonies tested, and the detection results of one colony are shown in Fig. S11. These results demonstrated that the inoculated KTU-TGVF cells thrived and accounted for the observed TCP degradation during a 30-day operating period in the bioreactor. In the future, the efficient TCP-mineralizing strain KTU-TGVF coupled with a field-scale reactor has enormous potential to be applied for the treatment of industrial wastewater containing TCP.

## Conclusions

In this work, an efficient TCP-mineralizing strain was created using combinatorial engineering strategies. In brief, we first integrated a synthetic pathway for aerobic TCP mineralization into the chromosome of *P. putida* KT2440 by functional assembly of various TCP-degrading enzymes from different bacteria. We then enhanced the utilization of TCP as a carbon source by eliminating the growth lag-phase of using GLY as a growth precursor. We additionally engineered the strain with the ability to mineralize TCP under oxygen-limited conditions through the heterologous expression of VHb. Lastly, we further improved the utilization of TCP by increasing intracellular ATP and NADPH levels resulting from deletion of the flagellar operon. We envision that these combinatorial engineering approaches could provide insights into devising engineering strategies to improve the degradation of anthropogenic chemicals by *P. putida*.

## Materials and Methods

### Reagents, strains, and culture conditions

TCP, DCP, epichlorohydrin (ECH), 3-chloropropane-1,2-diol (CPD) and GLY (99% pure analytical grade) were purchased from Alta Scientific Co., Ltd., Tianjin, China. All the other chemical reagents were of analytical grade and purchased from Dingguo Biotechnology Co. Ltd., Tianjin, China.

*E. coli* strains were grown at 37 °C in LB medium supplemented with 50 μg/ml kanamycin (Kan) if necessary. *P. putida* strains were grown at 30 °C in M9 minimal medium^16^ containing 0.5 mM TCP as well as in LB medium^32^ supplemented with 50 μg/ml Kan or 20 μg/ml 5-fluorouracil (5-FU) when required.

### DNA manipulation and strain constructions

Nine *P. putida* mutant strains with multiple gene insertions and/or deletions were constructed as described previously using a chromosomal scarless modification strategy^19^ and a suicide plasmid pK18mobsacB^33^. The detailed procedures for the construction of these nine *P. putida* mutant strains are described in Supplementary methods. The detailed information on the inserted exogenous genes (*dhaA90R*, *hheC*, *echA* and *vgb*) and deleted target genes (*glpR* and flagellar operon) is shown in Table 1. These four gene cassettes (*dhaA90R*, *hheC*, *echA* and *vgb*) were chemically synthesized by Genscript Co. Ltd., Nanjing, China and their nucleotide sequences are shown in Fig. S2. The strains, plasmids, and primers used in this study are listed in Table 2.

**Table 1:** Information on the heterologous genes and their chromosomal insertion sites in *P. putida* KT2440.

Gene	Length (bp)	Amino acid residues	Function	Strains (GenBank accession no.)	Insertion site/targeted deletion
*dhaA90R*	882	294	haloalkane dehalogenase	*Rhodococcus rhodochrous* NCIMB13064 (AF060871)	PP_1277 (*algA*)
*hheC*	765	255	haloalcohol dehalogenase	*Agrobacterium radiobacter* AD1 (AF397296)	PP_1278/PP_1279 (*algF/algJ*)
*echA*	885	295	epoxide hydrolase	*Agrobacterium radiobacter* AD1 (Y12804)	PP_1280 (*algI*)
*glpR*	756	252	glycerol-3-phosphate regulon repressor	*Pseudomonas putida* KT2440 (AE015451)	PP_1074
*vgb*	441	147	hemoglobin	*Vitreoscilla* sp. HG1 (AF292694)	PP_3356 (*fcs*)
*flagellar operon*	68139	69 genes	flagella synthesis	*Pseudomonas putida* KT2440 (AE015451)	PP_4329-PP_4397

**Table 2:** Strains, plasmids, and primers used in this study.

Strain, plasmid or primer	Relevant characteristics	Source or reference
Strains		
*E. coli*		
Trans1 T1	F^−^, φ80 (*lacZ*), ΔM15, Δ*lacX74*, *hsdR* (r_K_^−^, m_K_^+^), Δ*recA1398*, *endA1*, *tonA*	Transgen
*P. putida*		
KT2440	Wild type	ATCC 47054
KTU	*upp*-deficient KT2440	This study
KTU-T1	KT2440 mutant (Δ*upp*, Δ*algA*, *dhaA90R* ^*+*^)	This study
KTU-T2	KT2440 mutant (Δ*upp*, Δ*algF*&*algJ*, *hheC* ^+^)	This study
KTU-T3	KT2440 mutant (Δ*upp*, Δ*algI*, *echA* ^+^)	This study
KTU-T12	KT2440 mutant (Δ*upp*, ΔalgA, Δ*algF*&*algJ*, *dhaA90R* ^+^, *hheC* ^+^)	This study
KTU-T123	KT2440 mutant (Δ*upp*, Δ*algA*, Δ*algF*&*algJ*, Δ*algI*, *dhaA90R* ^+^, *hheC* ^+^, *echA* ^+^)	This study
KTU-TG	KT2440 mutant (Δ*upp*, Δ*algA*, Δ*algF*&*algJ*, Δ*algI*, Δ*glpR*, *dhaA90R* ^+^, *hheC* ^+^, *echA* ^+^)	This study
KTU-TGV	KT2440 mutant (Δ*upp*, Δ*algA*, Δ*algF*&*algJ*, Δ*algI*, Δ*glpR*, Δ*fcs*, *dhaA90R* ^+^, *hheC* ^+^, *echA* ^+^, *vgb* ^+^)	This study
KTU-TGF	KT2440 mutant (Δ*upp*, Δ*algA*, Δ*algF*&*algJ*, Δ*algI*, Δ*glpR*, Δflagellar operon, *dhaA90R* ^*+*^ *, hheC* ^*+*^ *, echA* ^*+*^)	This study
KTU-TGVF	KT2440 mutant (Δ*upp*, Δ*algA*, Δ*algF*&*algJ*, Δ*algI*, Δ*glpR*, Δ*fcs*, Δflagellar operon, *dhaA90R* ^*+*^ *, hheC* ^*+*^ *, echA* ^*+*^ *, vgb* ^*+*^)	This study
Plasmids		
pK18mobsacB	Kan^r^, suicide plasmid for gene knockout	33
pKU	Kan^r^, pK18mobsacB containing *upp* gene	This study
pKU-T1	Kan^r^, pK18mobsacB containing *upp* and *dhaA90R*	This study
pKU-T2	Kan^r^, pK18mobsacB containing *upp* and *hheC*	This study
pKU-T3	Kan^r^, pK18mobsacB containing *upp* and *echA*	This study
pKU-G	Kan^r^, pK18mobsacB containing *upp* and Δ*glpR*	This study
pKU-V	Kan^r^, pK18mobsacB containing *upp* and *vgb*	This study
pKU-F	Kan^r^, pK18mobsacB containing *upp* and Δflagellar operon	This study
Primers		
T1-1	5′-TTCTGGTGGTAGCGGTTCCCGTCTG-3'	This study
T1-2	5′-CTGCGCGATGGTCTTCACCGAAACG-3'	This study
T2-1	5′-GATGACCCTGCTCAACGGCAAGCTG-3'	This study
T2-2	5′-GGTCAGTTGCTGGTCGCTCAGGTTC-3'	This study
T3-1	5′-CCTGTTCCTGTTCTTGCCGATCTTC-3'	This study
T3-2	5′-CTGGAAGTAAAGGAACGGCGAGTAG-3'	This study
G-1	5′-CCCGAATTCCGAGTATTTCGCCAGCAAGG-3'	This study
G-2	5′-GCGGCATGCATGCCGGACAAATTCTGCAAT-3'	This study
G-3	5′-TTGTCCGGCATGCATGCCGCCAGACTTTTG-3'	This study
G-4	5′-CCCGAATTCAGGTGGTCGTAGAGGAACAG-3'	This study
V-1	5′-GCCCGGACACCACTTTCATC-3'	This study
V-2	5′-CTTGGGCATCGCGGTTCAAG-3'	This study
F-1	5′-AGACTTCCATTGCCAAAGCCCTCAC-3'	This study
F-2	5′-ACTGCGCGATGGTCTTCACCGAAAC-3'	This study
F-3	5′-AGACTTCCATTGCCAAAGCCCTCAC-3'	This study
F-4	5′-ACTGCGCGATGGTCTTCACCGAAAC-3'	This study
T1-F	5′-CAGCTACCTGTGGCGCAACATCATC-3'	This study
T1-R	5′-GTGCAGCCAGTTCATGTAGGCTTCC-3'	This study
T2-F	5′-CACGACGAGAGCTTCAAGCAGAAGG-3'	This study
T2-R	5′-AGAAGTACGGGCTGTCTTCGCTGTG-3'	This study
T3-F	5′-CCTGAGCAAGTACAGCCTGGACAAG-3'	This study
T3-R	5′-GTACTTCGGCACGAACTCGATCAGC-3'	This study
V-F	5′-ATGTTAGACCAGCAAACCATTAACA-3'	This study
V-R	5′-TTATTCAACCGCTTGAGCGTACAAA-3'	This study

Transformation of plasmid into *P. putida* was carried out using the electroporation method^34^. Screening of *P. putida* mutant strains was performed as described previously^19^. In brief, the single-crossover recombinants were screened by incubating at 30 °C for 24 h on LB agar plates supplemented with 50 μg/ml Kan. The selected recombinants were then incubated at 30 °C for 24 h in LB medium. To further screen the double-crossover recombinants, the culture broths that had been diluted to 10^−5^ were spread on LB agar plates supplemented with 20 μg/ml 5-FU. The selected recombinants showing 5-FU^r^ and Kan^s^ were further checked by PCR using primers listed in Table 2. All the constructed mutant strains were validated by DNA sequencing.

### RT-PCR assays

Total RNA was extracted from KTU-TGVF cells using a RNApure Bacteria Kit (CWBIO, China) and treated with DNase I at 37 °C for 30 min. RNA quality was checked by an Agilent 2100 Bioanalyzer. cDNA was synthesized with 0.5 μg total RNA as template using a PrimeScript RT Master Mix Kit (TaKaRa, Japan). PCR was carried out with cDNA as the template using PrimeSTAR HS DNA polymerase (TaKaRa) and primers listed in Table 2 on an ABI 2720 thermal cycler (Applied Biosystems). Genomic DNA, mRNA or ddH_2_O was used as the template in the control reactions. PCR products were detected by agarose gel electrophoresis.

### Resting-cell transformation assays

To verify the functionality of DhaA, HheC and EchA in *P. putida*, transformation assays were carried out with resting cells of KTU-T1, KTU-T2 or KTU-T3. Cells were pre-grown in M9 minimal medium containing 0.4% glucose at 200 rpm and 30 °C for 24 h, harvested by centrifugation, washed twice with 50 mM potassium phosphate buffer (pH 7.2), and resuspended (OD_600_ = 1.0) in the same buffer. Subsequently, resting-cell suspension was incubated with 0.2 mM TCP, DCP or ECH at 200 rpm and 30 °C. Samples were removed at 6 h for the analysis of transformation products by gas chromatography-mass spectrometer (GC-MS).

### TCP degradation studies

KTU-TGVF cells were inoculated at OD_600_ = 0.05 into 50 ml Erlenmeyer flasks containing 10 ml of M9 minimal medium supplemented with 0.5 mM TCP and samples were incubated in a shaking incubator at 200 rpm and 30 °C. Aliquots (1 ml) of samples were withdrawn at regular time intervals and extracted with equal volume of acetone. The mixture was centrifuged for 10 min at 4,000 rpm and 4 °C and the organic layer was recovered carefully. The extraction process was repeated three times. The organic layers were pooled and then dried over anhydrous Na_2_SO_4_. Then, the concentrations of TCP, DCP, ECH and CPD were measured by GC-MS. GLY was quantified using a free GLY assay kit (Sigma, USA).

Samples of 1 μl (diluted if necessary) were injected directly for GC-MS analysis, which was obtained on an Agilent Technologies 7890A-5975C (Agilent Technologies, Palo Alto, CA, USA). The GC was equipped with a HP-5 capillary column (30 m × 0.25 mm × 0.25 µm) and operated in split ratio of 20:1. Helium (>99.999%) was used as carrier gas with a constant flow rate of 0.6 ml/min. The GC conditions were as follows: injector temperature of 250 °C, oven temperature maintained at 35 °C for 6 min and then increased to 250 °C at a rate of 10 °C/min, and interface temperature of 230 °C. The mass spectrum conditions were as follows: ionization energy of 70 eV, scan range of 30–300 amu, and ion chamber temperature of 250 °C with tungsten filament used for ionization of molecules. The concentration of metabolites was determined using a standard curve of peak area versus concentration. For identification of metabolites, the mass spectra of metabolites were compared with those of their authentic standards or the same compound available in the National Institute of Standard Technology library, USA.

### VHb detection

VHb activity was detected by carbon monoxide (CO)-difference spectra. The complex consisting of CO and reduced hemoglobin has a characteristic peak at 420 nm^35^. KTU-TGVF or KTU cells were suspended in 50 mM potassium phosphate buffer (pH 7.2) and broken using an ultrasonic disruptor on ice (200 W for 15 min with cycles of sonication of 10 s each and 15 s pause). The crude extracts were centrifuged at 10,000 rpm and 4 °C for 15 min to remove cell debris. The soluble cellular fraction was exposed to CO for 2 min. Then, hemoglobin levels were determined by CO-difference spectra using a SHIMADZU UV-1800 spectrophotometer (SHIMADZU, Japan).

### Flagellum observation

For electron microscope, KTU-TGVF or KT2440 cells were negatively stained with 0.05% (w/v) uranyl acetate for 20 s on 200-mesh carbon-coated copper grids. Electron microscope images were captured using a JEOL JEM-1400 transmission electron microscope (TEM) (JEOL, Japan) operated at 80 kV and equipped with a GATAN Orius 832 CCD (GATAN, USA).

### Determination of the ATP/ADP and NADPH/NADP^+^ molar ratios

Cells were grown in M9 minimal medium containing 0.2% (w/v) glucose at 30 °C for 12 or 20 h. The intracellular levels of ADP and ATP were measured using an ADP/ATP ratio bioluminescence assay kit (BioVision, USA). The intracellular concentrations of NADP^+^ and NADPH were measured using an EnzyChrom™ NADP/NADPH assay kit (BioAssay Systems, USA).

### Biofilm formation assays

Biofilm formation was quantified using the crystal violet (CV) staining assay^30^. Cells were grown overnight at 30 °C and 170 rpm in M9 minimal medium supplemented with 0.4% (w/v) glucose. The OD_600_ of the cultures was adjusted to 0.05 before inoculating 200 μl into 96-well microtiter plates. The plates were incubated at 30 °C without shaking for 24 or 96 h, after which the culture broth was removed from the plates and the OD_600_ was measured to estimate the planktonic cells in the culture. The plates were washed three times with H_2_O to remove all non-adhered cells, and stained with 0.1% (w/v) CV for 30 min at room temperature. After discarding the dye, the plates were washed with H_2_O, dried and the remaining stain was dissolved in 33% (v/v) acetic acid. The plates were agitated gently for 1 h. Biofilm was quantified by measuring the absorbance at 590 nm (A_590_) using a BIOLOG microplate reader. The biofilm index was calculated as the ratio of biofilm formation to planktonic cell density (A_590_/OD_600_).

### Degradation of TCP by KTU-TGVF in a lab-scale bioreactor

A lab-scale reactor was designed for treatment of TCP-contaminated water (Fig. S10), which was mainly composed of a cylindrical vessel of polymethyl methacrylate with a volumetric capacity of 2 liters and a working volume of 0.8 liter. The reactor was filled with 500 g ceramic Raschig rings (outer diameter, 8 mm; internal diameter, 4 mm; length, 8 mm; Pingxiang Tower Packing Co.) as packing material. TCP-contaminated water was introduced into the reactor using a peristaltic pump from a glass vessel (0.5 liter) containing TCP in water. The airflow was regulated with a gas flow controller. The pH was controlled by adding 0.1 M NaOH when necessary.

For startup, the reactor was inoculated with a 800 ml suspension of cells grown on 0.5 mM TCP plus 0.4% glucose (OD_600_ = 0.4), which was left for 4 days without a supply of TCP-contaminated water. During this period, air was introduced into the reactor at a rate of 0.1 ml/min. After 4 days of inoculation, a wastewater stream containing TCP was introduced continuously, and the influent concentration of TCP was increased gradually from 0.05 to 0.2 mM. From day 18 onward, about 0.2 mM TCP was introduced into the reactor at a rate of 0.1 ml/min, and the flow rate was maintained during the operating period. The airflow was maintained at 1 ml/min to provide sufficient oxygen for aerobic mineralization of TCP. The effluent concentrations of TCP, DCP, and chloride ions were measured by GC-MS and a chloride assay kit (Sigma, USA) during a 30-day operating period.

## Electronic supplementary material

Supplemental material
