# Supplementary material for: Combinatorial metabolic engineering of Pseudomonas putida KT2440 for efficient mineralization of 1,2,3-trichloropropane
Source: Sci Rep. 2017 Aug 1;7:7064. doi: 10.1038/s41598-017-07435-x (PMC5539299; doi:10.1038/s41598-017-07435-x)
Supplement: Supplementary file 1 — Supplemental material [file 41598_2017_7435_MOESM1_ESM.doc]

**Supplemental material**

**Combinatorial metabolic engineering of *Pseudomonas putida* KT2440 for efficient mineralization of 1,2,3-trichloropropane**

Ting Gong,^1^ Xiaoqing Xu,^1^ You Che,^1^ Ruihua Liu,^2*^ Weixia Gao,^1^ Fengjie Zhao,^1^ Huilei Yu,^3^ Jingnan Liang,^4^ Ping Xu,^5^ Cunjiang Song,^1*^ Chao Yang^1*^

^1^Key Laboratory of Molecular Microbiology and Technology for Ministry of Education, Nankai University, Tianjin 300071, China

^2^State Key Laboratory of Medicinal Chemical Biology, Nankai University, Tianjin 300071, China

^3^State Key Laboratory of Bioreactor Engineering, East China University of Science and Technology, Shanghai 200237, China

^4^Core Facility of Equipment, Institute of Microbiology, Chinese Academy of Sciences, Beijing 100101, China

^5^State Key Laboratory of Microbial Metabolism, and School of Life Sciences & Biotechnology, Shanghai Jiao Tong University, Shanghai 200240, China

*Corresponding authors:

Ruihua Liu (Tel./fax: 86 22 2350 2351; E-mail: yangyangliu@nankai.edu.cn)

Cunjiang Song (Tel./fax: 86 22 2350 3866; E-mail: songcj@nankai.edu.cn)

Chao Yang (Tel./fax: 86 22 2350 3866; E-mail: yang_chao2008@hotmail.com)

**Fig. S1.** PCR detection of multiple gene insertions and deletions in *P. putida* KTU-TGVF using chromosomal DNA as template.

**Fig. S2.** The nucleotide sequences of four synthetic gene cassettes.

**Fig. S3.** Gene expression regulatory elements used in this work for the optimal expression of heterologous genes.

**Fig. S4.** RT-PCR assays for detecting transcription of heterologous genes in *P. putida* KTU-TGVF.

**Fig. S5.** Western blot analysis for expression of DhaA, HheC and EchA in *P. putida* KTU-TGVF.

**Fig. S6.** CO-difference spectral analysis for detection of VHb activity.

**Fig. S7.** Observation of flagellum by a transmission electron microscope.

**Fig. S8.** Growth curves of *P. putida* KTU-TGV and KTU-TGVF.

**Fig. S9.** Biofilm formation by *P. putida*.

**Fig. S10.** Schematic representation of a lab-scale reactor for treating TCP-contaminated water.

**Fig. S11.** PCR detection of gene insertions and deletions in the genome of the *P. putida* strain isolated from the reactor.

**Supplementary methods:** Construction of nine mutant strains of *P. putida* KT2440 by chromosomal scarless modification


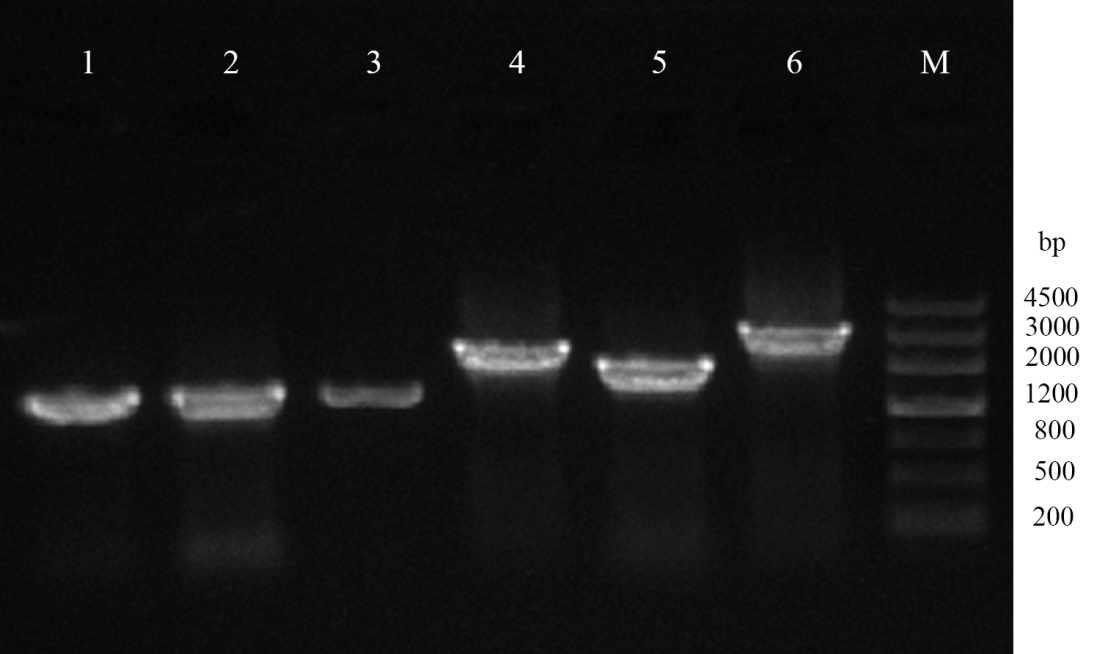


**Fig. S1.** PCR detection of multiple gene insertions and deletions in *P. putida* KTU-TGVF using chromosomal DNA as the template. Samples: lane M, DNA marker; lane 1, *dhaA90R*; lane 2, *hheC*; lane 3, *echA*; lane 4, *vgb*; lane 5, ∆*glpR*; lane 6, ∆flagellar operon.

**(A)**

GAATTCATACGCTGTTCCAGCAAACCATCGAGCGCCTGGTATTCGAGGGCATGGACACCCCGATCGTGGTCTGTAACAAGGACCACAAGTTCATCGTCCAGGAGCAACTGGCCGCACTGAAGCTGGAAACCCAAGGCATCCTCATGGAACCGTTCGGCCGCAACACCGCGCCGGCCGTGGCCATGGCTGCCATGAAGCTGGTCAACGAAGGCCGCGACGAGCTGATGCTGGTGCTGCCTGCCGATCATGTGATCGATGACCAGAAAGCCCTGCAACGTGCCTTGGCCTTGGCCACCGTGGCCGCCGAGCGTGGCGAGATGGTGCTGTTCGGCGTGCCGGCGACCAAGCCGGAAACCGGCTACGGCTACATCCGTTCCAGCCAGGATGCGCTGCTGCCCGAAGGCGTGGCGCGGGTTGCGCAGTTCGTCGAGAAGCCCGACGAGAAACGCGCGGCCGAGTTCGTCCAGGCCGGTGGTTACTTCTGGAACAGCGGTATGTTCCTGTTCTTAATTAAGCCCATTGACAAGGCTCTCGCGGCCAGGTATAATTGCACGACCTAGGGCCCAAGTTCACTTAAAAAGGAGATCAACAATGAAAGCAATTTTCGTACTGAAACATCTTAATCATGCTAAGGAGGTTTTCTAATGTCGGAAATCGGCACCGGCTTCCCGTTCGACCCGCACTACGTGGAAGTCCTGGGCGAACGCATGCACTATGTCGACGTGGGCCCGCGCGACGGCACCCCGGTGCTGTTTCTGCATGGCAACCCGACCAGCTCGTACCTGTGGCGCAACATCATCCCGCACGTGGCCCCGAGCCATCGCTGTATCGCGCCGGACTTGATCGGCATGGGTAAGAGCGACAAGCCGGACCTGGACTACTTCTTCGACGACCACGTGCGCTATCTGGACGCCTTCATCGAGGCCCTGGGCCTGGAGGAAGTGGTCCTGGTGATCCACGACTGGGGTAGCGCGCTGGGCTTCCACTGGGCCAAGCGTAATCCGGAACGCGTGAAGGGCATCGCCTGCATGGAGTTCATCCGCCCGCTGACCACCTGGGACGAATGGCCGGAGTTCGCCCGCGAAACCTTCCAGGCGTTTCGTACCGCCGACGTGGGCCGCGAGCTGATCATCGACCAGAACATGTGGATCGAGGGCCTGATTCCCGCGGGTGTGATCCGCCCGCTGACCGAAGTGGAGATGGACCACTACCGCGAGCCGTTCCTGAAACCGGTGGACCGCGAACCGCTGTGGCGCTTCCCGAACGAGCTGCCGATTGCCGGCGAACCCGCCAACATCGTGGCCCTGGTGGAGGCCTACATGAACTGGCTGCACCAGAGCCCGGTGCCGAAGCTGCTGTTCTGGGGCAACCCGGGCTATCTGATCACCCCGGCCGAAGCCGCCCGCCTGGCCGAGTCGCTGCCGAACTGCAAGACCGTGGATATCGGCCCGGGCTTGCATTTCCTGCAGGAGGACAACCCGGACCTGATCGGCAGCGAAATCGCGCGCTGGTTGCCCGCCCTGTGACTTGGACTCCTGTTGATAGATCCAGTAATGACCTCAGAACTCCATCTGGATTTGTTCAGAACGCTCGGTTGCCGCCGGGCGTTTTTTATTGGTGAGAATCCAGAACGGCAACGTCACCAAGGGCGACGTGGTGGTTCAAGACAGCCGCAACTGCATGATCCACGGCAACGGCAAACTGGTGTCGGTGATCGGCCTGGAGAACATCGTGGTGGTCGAGACCAAGGATGCCATGATGATTGCCCACAAGGACAAGGTCCAGGGCGTCAAGCAGATGGTCAAGACCCTCGACGAACAGGGCCGCACGGAAACCCAGAACCACCTGGAAGTGTATCGCCCGTGGGGCTCGTACGACTCGGTGGACATGGGCGGCCGCTTCCAGGTCAAGCACATCACCGTCAAGCCGGGCGCCAGCCTCTCGCTGCAGATGCACCACCACCGCGCCGAACACTGGATCGTGGTATCCGGTACCGCCGAGGTGACCTGCGACGAGAACGTGTTCTTGCTGACCGAAAACCAGTCGACCTACATCCCCATCGCTTCGGTGCACCGTTTGCGCAACCCGGGCAAGATCCCGCTGGAGATCATCGAGGTGCAGTCCGGGAGGAATTC

**(B)**

TTTGAATTCCAGATGACCCTGCTCAACGGCAAGCTGGCCAAGGCCGCCGAGACCCACTACGACGCCGAGTTCCCGATCAAGCGCCTGGGCACCAACGTGTGGGCGGCCATGGACTTCAAGCTGTTCAACGAAGGCCGCCCAGGTGTGGTACTGGGCCGCGACCAGTGGTTGTTCAGCGACGAAGAGTTCAAGCCCACCGCCGGTGCCGAGCAATTGATGCAGGAAAACCTGGCACTGATCCGTGGCGTACGCGACACCCTGCAGCAACACGGTAGCCAGCTGGTGCTGGCGATCGTGCCGGCCAAGGCACGGGTCTACACCGAGTACCTGGGCAAAGAGCGGCCTGCCAGCCTGCATGACGACCTGTACAACCAGTTCCATGCCCAGGCACGCCAGGCCAACGTGTTCGCACCGGACCTGATGGCACCGATGGAGCAGGCCAAGGCCCGCGGCCAGGTATTCCTGCGTACCGATACCCACTGGACGCCTATGGGTGCCGAAGTGGCGGCGCAGGCGCTGGCCGAAGCGGTCAGCCGCCAGAGCCTGCTCAACGGCGACCCACAAGCCTTCATCACCGAAGCCGGCAACACCGCCCCCTACAAGGGCGACTTAATTAAGCCCATTGACAAGGCTCTCGCGGCCAGGTATAATTGCACGACCTAGGGCCCAAGTTCACTTAAAAAGGAGATCAACAATGAAAGCAATTTTCGTACTGAAACATCTTAATCATGCTAAGGAGGTTTTCTAATGAGCACCGCCATCGTGACCAACGTGAAGCACTTCGGCGGCATGGGCAGCGCCCTGCGCCTGAGCGAGGCCGGCCACACCGTGGCCTGCCACGACGAGAGCTTCAAGCAGAAGGACGAGCTGGAAGCCTTCGCCGAGACCTACCCGCAGCTGAAGCCGATGAGCGAGCAGGAACCGGCCGAGCTGATCGAAGCCGTGACCAGCGCCTACGGCCAGGTGGACGTGCTGGTCAGCAACGACATCTTCGCCCCGGAGTTCCAGCCGATCGACAAGTACGCCGTGGAGGACTACCGCGGCGCCGTGGAGGCCCTGCAAATCCGCCCGTTCGCCCTGGTGAACGCCGTGGCCAGCCAGATGAAGAAGCGCAAGAGCGGCCACATCATCTTCATCACCAGCGCCACCCCGTTCGGCCCGTGGAAGGAGCTGAGCACCTACACCAGCGCCCGTGCCGGCGCCTGCACCCTGGCCAACGCCCTGAGCAAGGAGCTGGGCGAATACAACATCCCGGTGTTCGCCATCGGCCCGAACTACCTGCACAGCGAAGACAGCCCGTACTTCTACCCGACCGAGCCGTGGAAGACCAACCCGGAACACGTGGCCCACGTGAAGAAGGTGACCGCCCTGCAACGCCTGGGCACCCAGAAGGAGCTGGGCGAACTGGTGGCCTTCCTGGCCAGCGGCAGCTGCGACTACCTGACCGGCCAGGTGTTCTGGCTGGCCGGCGGCTTCCCGATGATCGAGCGCTGGCCGGGCATGCCGGAATGACTTGGACTCCTGTTGATAGATCCAGTAATGACCTCAGAACTCCATCTGGATTTGTTCAGAACGCTCGGTTGCCGCCGGGCGTTTTTTATTGGTGAGAATCCAGCCCGAACGTTATCTGCCAATGAAAAACGACCTCAGCAGCTTCGATCCGCAGTGGATCGCGCAGCTGAAAAACTCCCGTAAATCCGAAGAAAACCTGGCCTTGTCGTCCACCCGGACAGACCACTGACTGATAGAGAGGAAACGCACATGACTACCAAGACTTCCATTGCCAAAGCCCTCACCCTCGCGGCCGGCCTTTCCCTTGCTTCGATGCAGGCCTTCGCTGGTGCCGACGCCGCACTGTATGGCCCAAGCGCGCCGAAAGGCTCGACCTTCGTACGCCTGTACAACGCGACCAGCGCACCGGCCGCCGCGTCGGTCGGCAACACCCAGATCAAACAGGTGGGCGCACAGGCCAGCAGCGACTTCAGCTTCCTGCCAGGCGGCGACTACACCGCCCAGGTCGGCGGCAAGAGCGTGCCGGTCAAGCTGGCCTCGGACAAGTACTACACCCTGGTCAACAGCAACAGCGGCAGCCCGAAACTGATCGAAGAACCACCGTTCAAGAACAAGCAGAAAGCCCTGGTGCGCGTGCAGAACCTGAGCGACCAGCAACTGACCCTGAAAACCGCCGACGGCAAGACCGAAGTGGTCAAGCCGGGAATTCTTT

**(C)**

TTTGAATTCATGGTCTTCTCGTCCAACGTGTTCCTGTTCCTGTTCTTGCCGATCTTCCTCGGCCTGTACTACTTGAGCGGGCAACGTTATCGCAACCTGCTGCTGCTGGTCGCCAGCTACATCTTCTACGCCTGGTGGCGGGTGGACTTCCTGGCACTGTTCGCCGGCGTCACCCTGTGGAACTACTGGATCGGCCTGAAAGTCGGTGCCGCCGGTGTGCGCACCAAGCCTGCGCAGCGCTGGCTGCTGCTCGGCGTGGGTGTCGACCTGGCAATCCTCGGCTACTTCAAGTACGCCAACTTCGGCGTCGACAGCCTGAACGCGATCATGACGTCGTTTGGCCTGGAGCCATTCATTCTGACCCACGTGCTGCTGCCGATCGGTATCTCGTTCTACATCTTCGAGTCGATCAGCTACATCATCGACGTATACCGCGGTGACACCCCGGCTACCCGCAACCTGATCGACTTCGCAGCGTTCGTGGCGATTTTCCCGCACCTGATCGCAGGCCCCGTGCTGCGCTTCAAGGATTTGGTCGACCAGTTCAACAACCGCACCCACACCCTGGACAAGTTCTCCGAAGGCTGCACCCGCTTCATGCAGGGCTTCTTAATTAAGCCCATTGACAAGGCTCTCGCGGCCAGGTATAATTGCACGACCTAGGGCCCAAGTTCACTTAAAAAGGAGATCAACAATGAAAGCAATTTTCGTACTGAAACATCTTAATCATGCTAAGGAGGTTTTCTAATGACCATCCGTCGCCCGGAAGACTTCAAGCACTACGAGGTGCAGCTGCCGGACGTGAAGATCCACTACGTGCGCGAAGGCGCCGGCCCGACCCTGCTGCTGCTGCACGGCTGGCCGGGCTTCTGGTGGGAATGGAGCAAGGTGATCGGCCCGCTGGCCGAGCACTACGACGTGATCGTGCCGGACCTGCGCGGCTTCGGCGACAGCGAAAAGCCGGACCTGAACGACCTGAGCAAGTACAGCCTGGACAAGGCCGCCGACGACCAGGCCGCCCTGCTGGACGCCCTGGGCATCGAGAAGGCCTACGTGGTGGGCCACGACTTCGCCGCCATCGTGCTGCACAAGTTCATCCGCAAGTACAGCGACCGCGTGATCAAGGCCGCCATCTTCGACCCGATCCAGCCGGACTTCGGCCCGGTGTACTTCGGCCTGGGCCACGTGCACGAAAGCTGGTACAGCCAGTTCCACCAGCTGGACATGGCCGTGGAAGTGGTGGGCAGCAGCCGCGAGGTGTGCAAGAAGTACTTCAAGCACTTCTTCGACCACTGGAGCTACCGCGACGAACTGCTGACCGAGGAAGAGCTGGAGGTGCACGTGGACAACTGCATGAAGCCGGACAACATCCACGGCGGCTTCAACTACTACCGCGCCAACATCCGCCCGGACGCCGCCCTGTGGACCGACCTGGACCACACCATGAGCGACCTGCCGGTGACCATGATCTGGGGCCTGGGCGACACCTGCGTGCCGTACGCCCCGCTGATCGAGTTCGTGCCGAAGTACTACAGCAACTACACGATGGAGACCATCGAGGACTGCGGCCACTTCCTGATGGTGGAAAAGCCGGAGATCGCCATCGACCGCATCAAGACCGCCTTCCGCTGACTTGGACTCCTGTTGATAGATCCAGTAATGACCTCAGAACTCCATCTGGATTTGTTCAGAACGCTCGGTTGCCGCCGGGCGTTTTTTATTGGTGAGAATCCAGACCTGGCTGCGCGACTATCTGTACATCACCCTGGGCGGTAACCGCAAAGGCACCTTCAATACCTACCGCAACCTGTTCCTGACCATGCTGCTGGGCGGCCTGTGGCACGGTGCAAACTTCACCTACATCATCTGGGGCGCCTGGCACGGCATGTGGCTGGCCATCGAGCGCGCGTTGGGCCTCGACACCAACCCGCAGCGCTTCAACCCGGTGAAGTGGGCCTTCACCTTCCTGCTGGTGGTAGTCGGTTGGGTGATCTTCCGCGCCGAAAACCTGCATGTGGCCGCCCGTATGTACGGCGCGATGTTCAGCTTCGGCGACTGGCAGCTGTCGGAGCTCAACCGCGCCCAGCTCACCGGTCTGCAGGTGGCCACCCTGGTCATCGCCTACCTCACCCTGGCGTTCTTCGGCCTGCGCGACTTCTACCGCAATGCCAGGCCGACGCCCAAGGCGACCCCGGTGCAGGTCAACGCTGACGGCTCGATCGGCCTGGACTGGACCCGGGTGATGACCCGCGCACTGATCCTGCTGCTGTTCGTCGCCTCGATCCTCAAGCTTTCGGCGCAAAGCTACTCGCCGTTCCTTTACTTCCAGTTCTGAGAATTCTT

**(D)**

TTTGAATTCATGCAACCTGTCGAGCCACTGGCGCCGCTGCCGGCGCGCCTGCTCGAGCGCCTGGTGCATTGGGCCCAGGTGCGCCCGGACACCACTTTCATCGCGGCACGCCAGGCAGACGGTGCCTGGCGTTCGATCAGCTACGTGCAGATGCTCGCCGATGTGCGCACCATCGCCGCCAACTTGCTAGGACTGGGCCTCAGTGCCGAGCGCCCGCTGGCGCTGCTTTCCGGCAACGACATCGAACACCTGCAAATCGCCCTCGGCGCCATGTATGCCGGTATTGCCTATTGCCCGGTGTCGCCGGCCTACGCGCTGTTGTCGCAAGACTTCGCCAAGTTGCGCCATGTCTGCGAGGTGCTCACCCCCGGAGTGGTCTTCGTCAGCGACAGCCAGCCGTTCCAGCGCGCCTTCGAGGCGGTGCTGGACGATTCGGTCGGCGTGATCAGCGTGCGTGGCCAGGTCGCAGGTCGCCCCCATATAAGCTTCGACAGCCTGTTGCAACCGGGTGACCTGGCGGCGGCCGATGCGGCTTTCGCCGCCACCGGGCCGGACACCATCGCCAAATTCCTCTTCACCTCGGGCTCGACCAAGCTGCCCAAGGCGGTGTTAATTAAGCCCATTGACAAGGCTCTCGCGGCCAGGTATAATTGCACGACCTAGGGCCCAAGTTCACTTAAAAAGGAGATCAACAATGAAAGCAATTTTCGTACTGAAACATCTTAATCATGCTAAGGAGGTTTTCTAATGTTAGACCAGCAAACCATTAACATCATCAAAGCCACTGTTCCTGTATTGAAGGAGCATGGCGTTACCATTACCACGACTTTTTATAAAAACTTGTTTGCCAAACACCCTGAAGTACGTCCTTTGTTTGATATGGGTCGCCAAGAATCTTTGGAGCAGCCTAAGGCTTTGGCGATGACGGTATTGGCGGCAGCGCAAAACATTGAAAATTTGCCAGCTATTTTGCCTGCGGTCAAAAAAATTGCAGTCAAACATTGTCAAGCAGGCGTGGCAGCAGCGCATTATCCGATTGTCGGTCAAGAATTGTTGGGTGCGATTAAAGAAGTATTGGGCGATGCCGCAACCGATGACATTTTGGACGCGTGGGGCAAGGCTTATGGCGTGATTGCAGATGTGTTTATTCAAGTGGAAGCAGATTTGTACGCTCAAGCGGTTGAATAACTTGGACTCCTGTTGATAGATCCAGTAATGACCTCAGAACTCCATCTGGATTTGTTCAGAACGCTCGGTTGCCGCCGGGCGTTTTTTATTGGTGAGAATCCAGGGCCCGCATATCATGCCGGGCTACTGGCGCTCGCCGCAGCAGACCGCCGAGGCGTTCGACGAGGAGGGCTTCTACTGTTCGGGCGACGCGTTGAAGCTGGCCGATGCCAGGCAGCCCGAGCTTGGCCTGATGTTCGATGGCCGTATCGCTGAGGACTTCAAACTTTCGTCCGGGGTATTCGTCAGTGTCGGGCCGCTGCGCAACCGCGCAGTGCTGGAGGGCTCGCCTTACGTACAGGACATCGTGGTCACCGCGCCGGACCGTGAATGCCTGGGCCTGCTGGTGTTCCCGCGTCTGCCCGAGTGTCGGCGCCTGGCCGGGCTGGCAGAGGATGCCAGCGATGCGCGGGTGCTGGCCAACGACACCGTGCGCAGTTGGTTCGCTGACTGGCTGGAGCGCTTGAACCGCGATGCCCAAGGCAACGCCAGCCGTATCGAATGGCTGTCGCTGCTGGCCGAGCCGCCGTCGATCGACGCCGGTGAAATCACCGACAAGGGCTCGATCAATCAGCGCGCCGTGCTGCAGCGGCGCGCCGCTCAGGTCGAGGCGCTGTACCGTGGCGAAGACCCCGACGCATTGCACGCCAAGGTGCGGCCTTGAGAATTCTTT

**Fig. S2.** The nucleotide sequences of four synthetic gene cassettes. (A) *dhaA90R* gene cassette; (B) *hheC* gene cassette; (C) *echA* gene cassette; (D) *vgb* gene cassette.


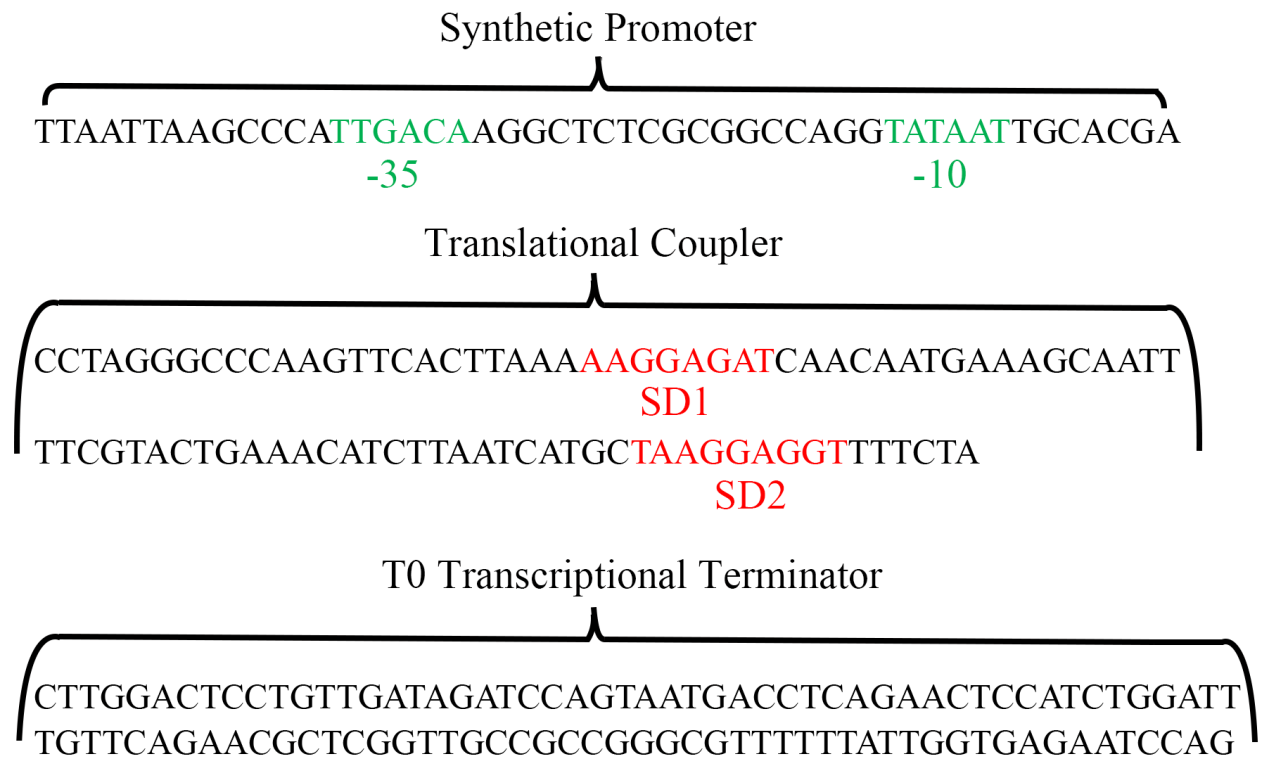


**Fig. S3.** Gene expression regulatory elements used in this work for the optimal expression of heterologous genes.


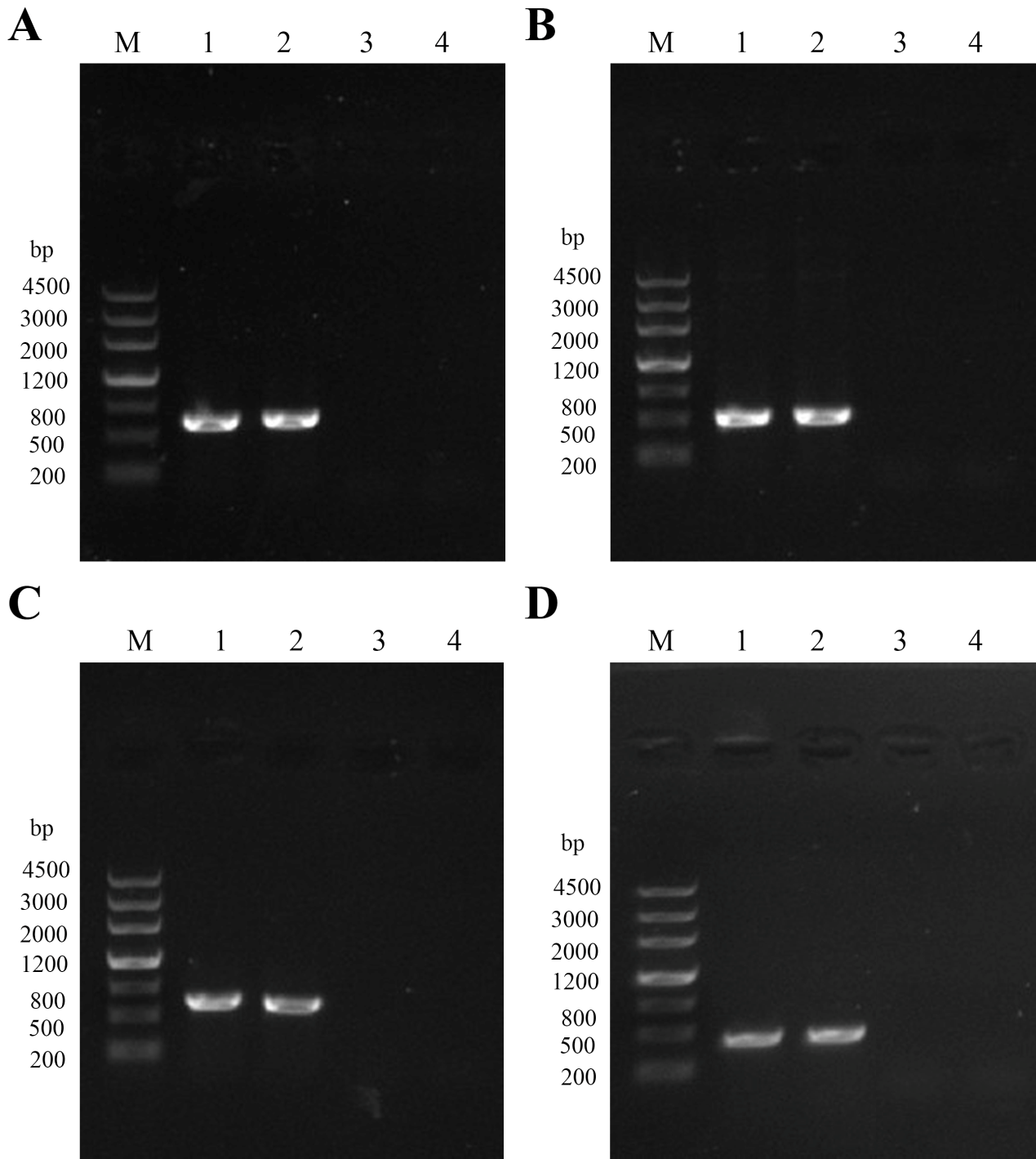


**Fig. S4.** RT-PCR assays for detecting transcription of heterologous genes in *P. putida* KTU-TGVF. Panels A-D are the detection results of *dhaA90R*, *hheC*, *echA* and *vgb*, respectively. Samples: lane M, DNA marker; lane 1, genomic DNA; lane 2, cDNA; lane 3, mRNA; lane 4, ddH_2_O.


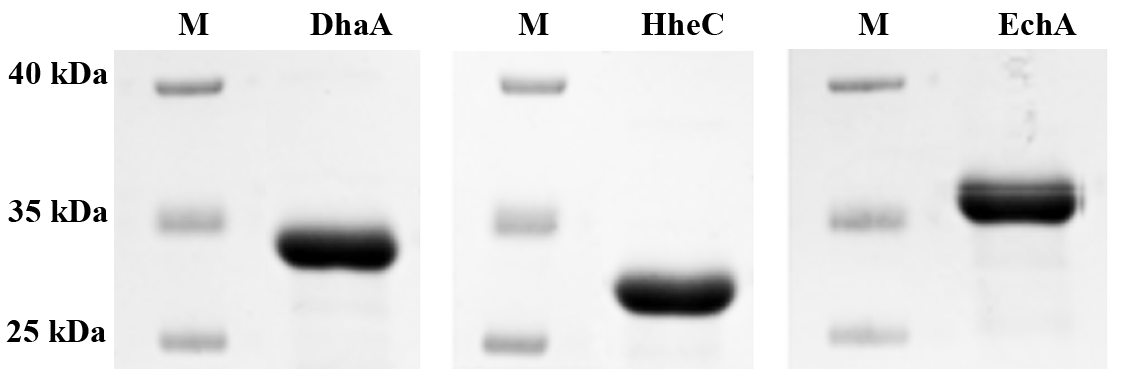


**Fig. S5.** Western blot analysis for expression of DhaA, HheC and EchA in *P. putida* KTU-TGVF. Antibodies to DhaA, HheC and EchA were used at a 1:500 dilution. Horseradish peroxidase-conjugated IgG antibody was used at a 1:2,000 dilution. Immunoreactive bands were detected by enhanced chemiluminescence using an ECL Plus kit. The theoretical molecular weights of DhaA, HheC and EchA are 34 kDa, 29 kDa and 35 kDa, respectively.


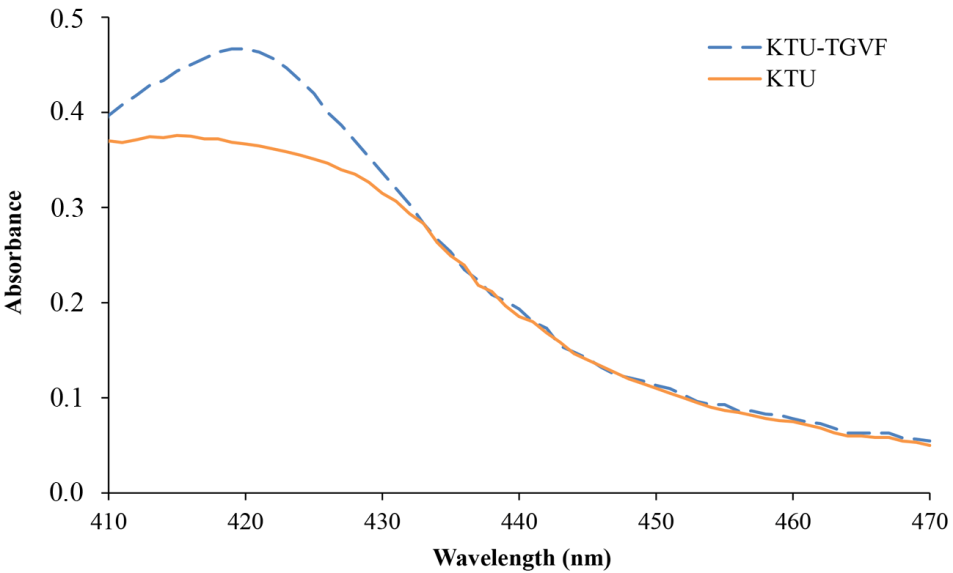


**Fig. S6.** CO-difference spectral analysis for detection of VHb activity. Cell extracts from *P. putida* KTU-TGVF showed a typical VHb-CO binding absorption spectrum with a characteristic peak at 420 nm, whereas the peak was absent in CO binding absorption spectrum of cell extracts from *P. putida* KTU.


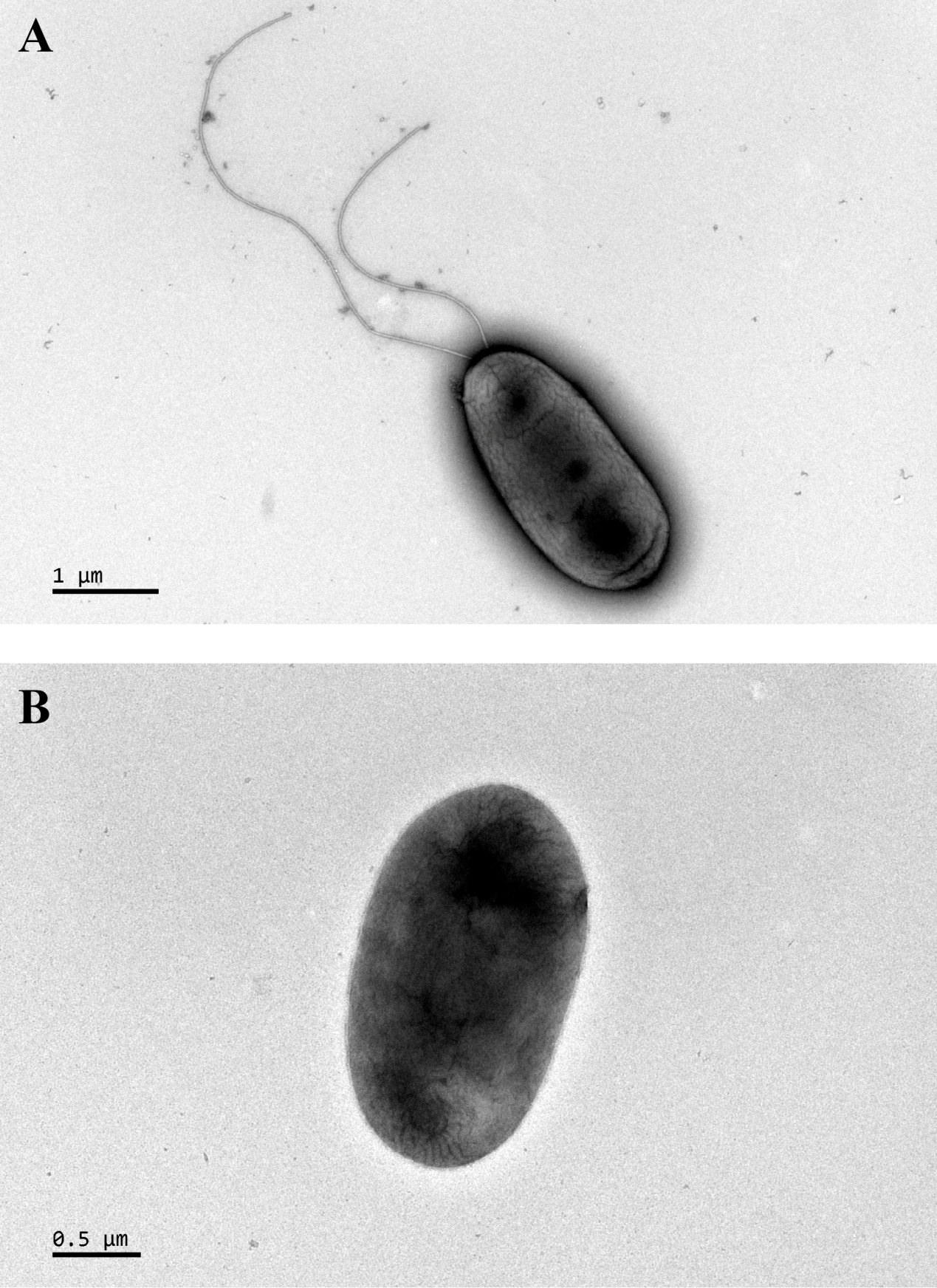


**Fig. S7.** Observation of flagellum by a transmission electron microscope. (A) *P. putida* KT2440, (B) *P. putida* KTU-TGVF.


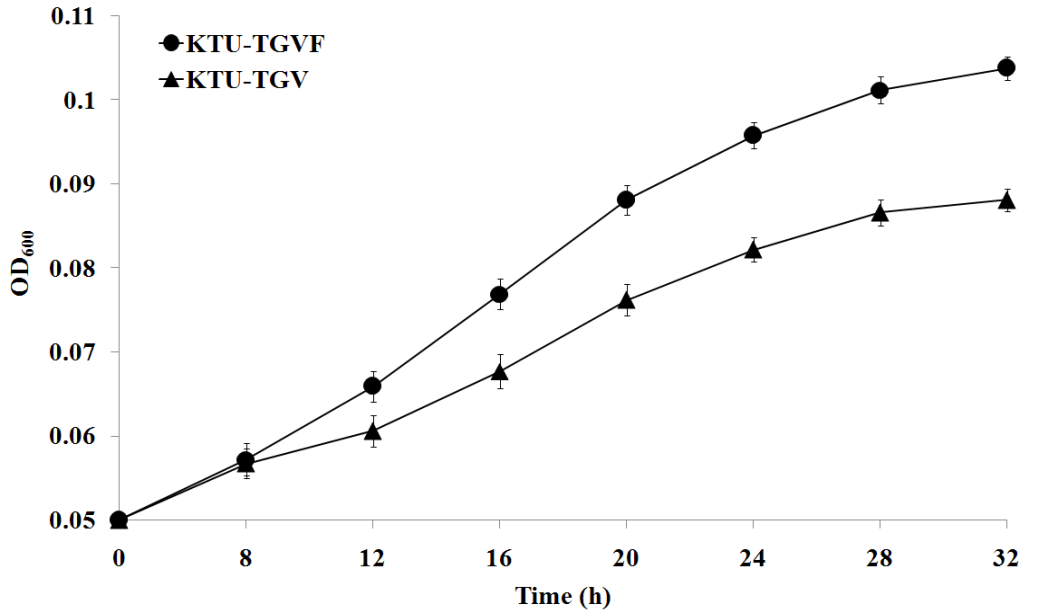


**Fig. S8.** Growth curves of *P. putida* KTU-TGV and KTU-TGVF. Cells were grown in M9 minimal medium supplemented with 0.5 mM TCP in a shaking incubator at 200 rpm and 30°C and the OD_600_ was measured to estimate cell growth. Bars represent the mean values ± standard deviation of triplicate measurements from three independent experiments.


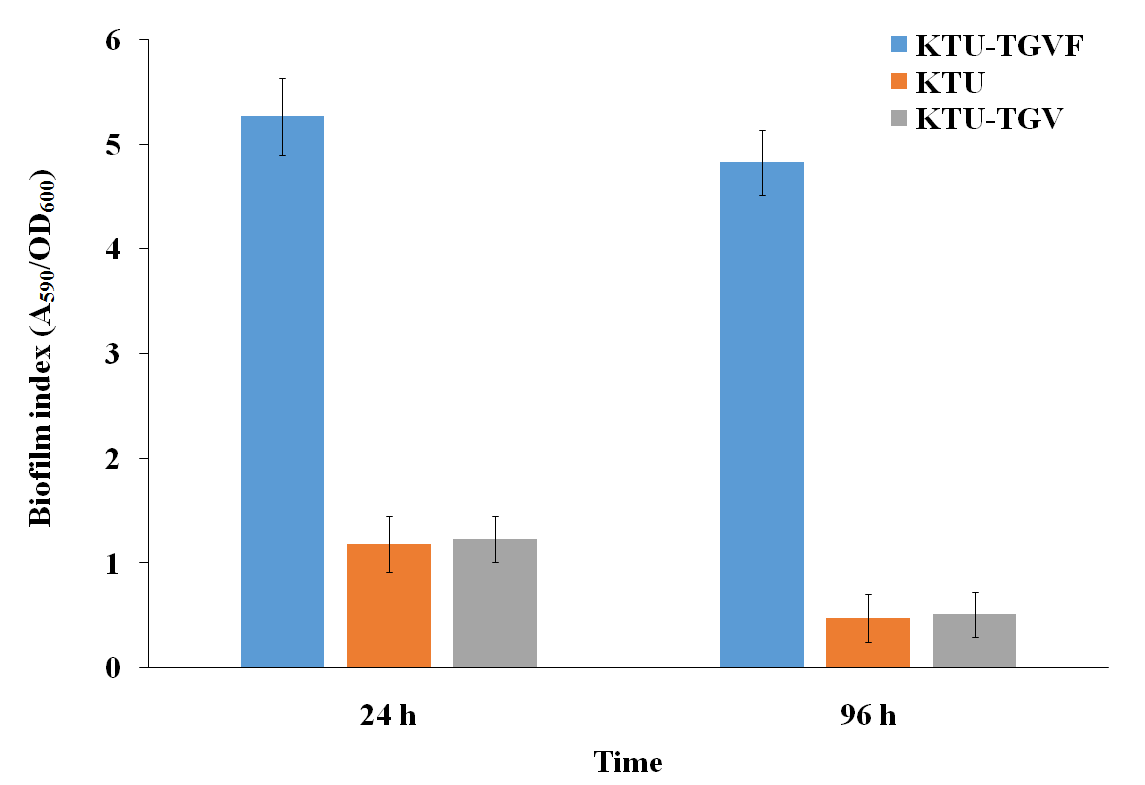


**Fig. S9.** Biofilm formation by *P. putida*. Cells were grown in M9 minimal medium containing 0.4% (w/v) glucose without shaking for 24 or 96 h at 30°C, after which the culture broth was removed and biofilm was visualized by staining the attached cells with CV as described in Materials and methods. The biofilm index was calculated by normalizing the absorbance of CV at 590 nm (A_590_) to planktonic cell density (OD_600_). Bars represent the mean values ± standard deviation of triplicate measurements from three independent experiments.


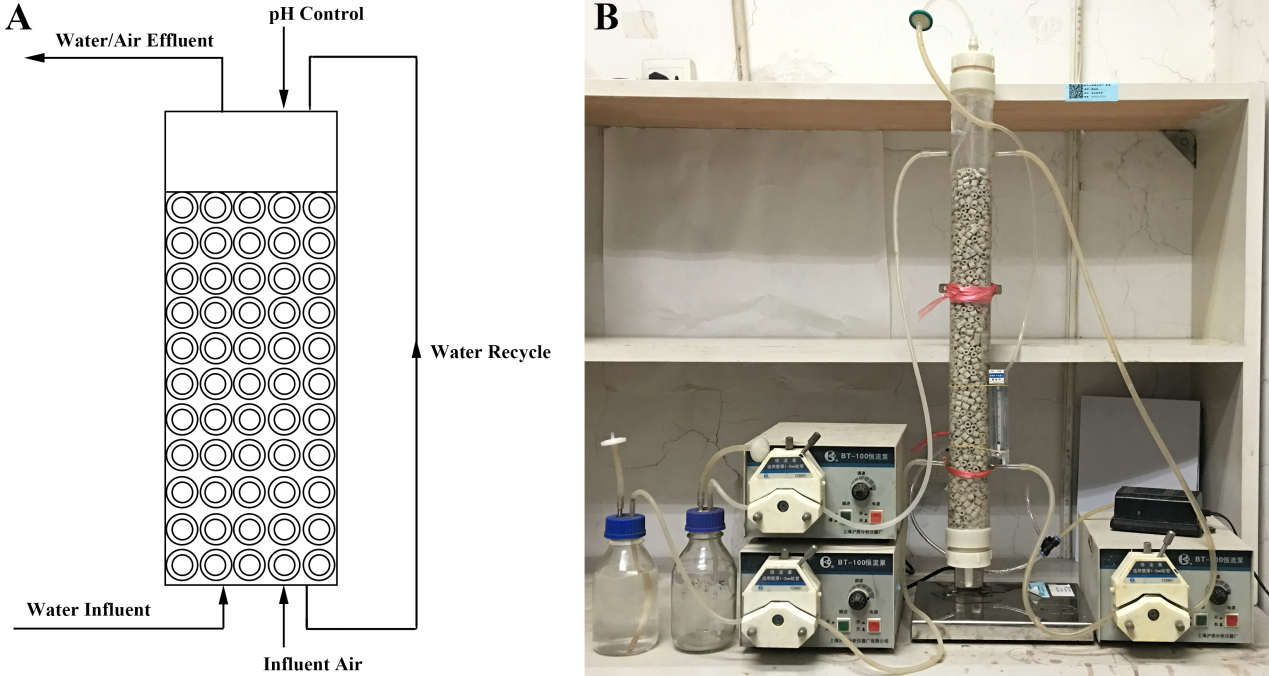


**Fig. S10.** Schematic representation of a lab-scale reactor for treating TCP-contaminated water. The reactor contained *P. putida* KTU-TGVF cells immobilized on ceramic rings and was continuously operated for 30 d with the TCP influent concentration of about 0.2 mM.


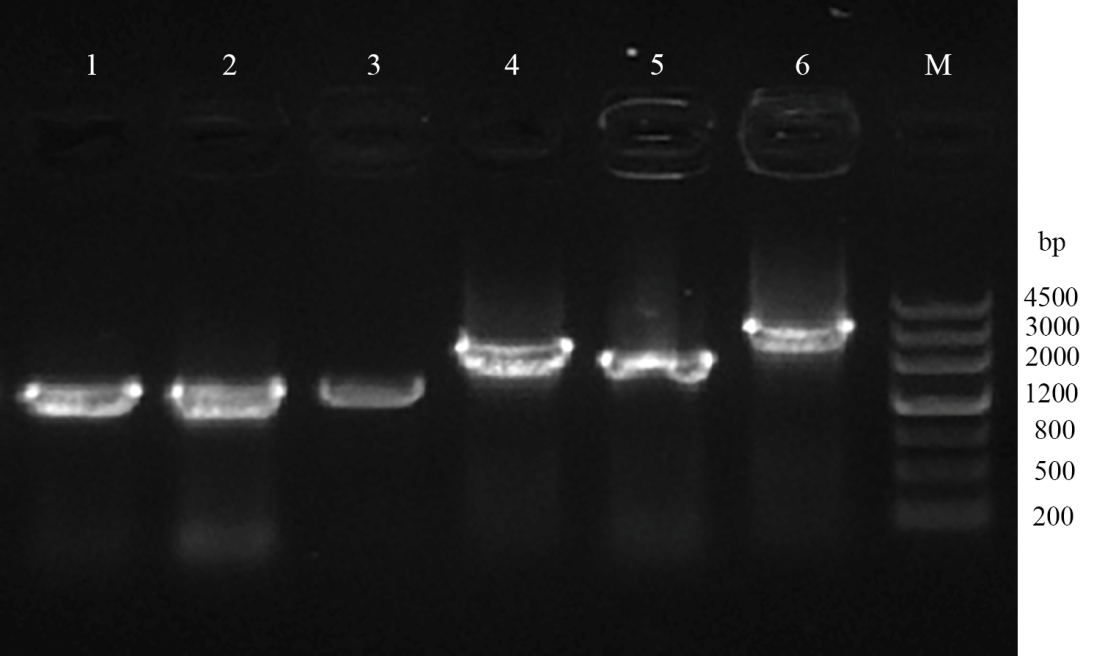


**Fig. S11.** PCR detection of gene insertions and deletions in the genome of the *P. putida* strain isolated from the reactor. Samples: lane M, DNA marker; lane 1, *dhaA90R*; lane 2, *hheC*; lane 3, *echA*; lane 4, *vgb*; lane 5, ∆*glpR*; lane 6, ∆flagellar operon.

**Supplementary methods:** Construction of nine mutant strains of *P. putida* KT2440 by chromosomal scarless modification

*Construction of P. putida KTU-T1 by chromosomal scarless modification.* A gene cassette containing the upstream and downstream homologous arms, a *P. putida* strong promoter, two SD sequences, a haloalkane dehalogenase gene (*dhaA90R*), and a T0 transcriptional terminator was chemically synthesized by Genscript, Nanjing, China. The nucleotide sequence of this synthetic gene cassette is shown in Fig. S1. The synthetic gene cassette was released with *Eco*RI from pCCI vector and then subcloned into the same restriction site of pKU to generate the gene targeting vector pKU-T1.

Plasmid pKU-T1 was transformed into *P. putida* KTU using the electroporation method and then integrated into the chromosome of *P. putida*. Firstly, the recombinants occurring with the first crossover were selected on LB agar plates containing 50 μg/ml kanamycin (LB_Kan_). Furthermore, the recombinants occurring with the second crossover were screened on LB agar plates supplemented with 20 μg/ml 5-FU (LB_5-FU_). Those recombinants showing 5-FU^r^ and Kan^s^ were further checked by PCR and DNA sequencing. The resulting mutant was designated as *P. putida* KTU-T1.

*Construction of P. putida KTU-T2 and KTU-T12 by chromosomal scarless modification.* A gene cassette containing the upstream and downstream homologous arms, a *P. putida* strong promoter, two SD sequences, a halohydrin dehalogenase gene (*hheC*), and a T0 transcriptional terminator was chemically synthesized by Genscript, Nanjing, China. The nucleotide sequence of this synthetic gene cassette is shown in Fig. S1. The synthetic gene cassette was released with *Eco*RI from pCCI vector and then subcloned into the same restriction site of pKU to generate the gene targeting vector pKU-T2.

Plasmid pKU-T2 was transformed into *P. putida* KTU and KTU-T1 using the electroporation method. Screening of the mutant strains was carried out as described above. The resulting mutant strains were designated as *P. putida* KTU-T2 and KTU-T12.

*Construction of P. putida KTU-T3 and KTU-T123 by chromosomal scarless modification.* A gene cassette containing the upstream and downstream homologous arms, a *P. putida* strong promoter, two SD sequences, an expoxide hydrolase gene (*echA*), and a T0 transcriptional terminator was chemically synthesized by Genscript, Nanjing, China. The nucleotide sequence of this synthetic gene cassette is shown in Fig. S1. The synthetic gene cassette was released with *Eco*RI from pCCI vector and then subcloned into the same restriction site of pKU to generate the gene targeting vector pKU-T3.

Plasmid pKU-T3 was transformed into *P. putida* KTU and KTU-T12 using the electroporation method. Screening of the mutant strains was carried out as described above. The resulting mutant strains were designated as *P. putida* KTU-T3 and KTU-T123.

*Construction of P. putida KTU-TG by chromosomal scarless modification.* The upstream and downstream fragments of *glpR* gene were obtained by PCR with Phanta HS Super-Fidelity DNA Polymerase (Vazyme Biotech, Nanjing, China) using primers G-1/G-2 and G-3/G-4, respectively. Subsequently, the two fragments were ligated together using overlap PCR. The generated fragment was digested with *Eco*RI and then subcloned into the same restriction site of pKU to create the gene knockout vector pKU-G.

Plasmid pKU-G was transformed into *P. putida* KTU-T123 using the electroporation method. Screening of the mutant strain was carried out as described above. The resulting mutant strain was designated as *P. putida* KTU-TG.

*Construction of P. putida KTU-TGV by chromosomal scarless modification.* A gene cassette containing the upstream and downstream homologous arms, a *P. putida* strong promoter, two SD sequences, a *Vitreoscilla* hemoglobin gene (*vgb*), and a T0 transcriptional terminator was chemically synthesized by Genscript, Nanjing, China. The nucleotide sequence of this synthetic gene cassette is shown in Fig. S1. The synthetic gene cassette was released with *Eco*RI from pCCI vector and then subcloned into the same restriction site of pKU to generate the gene targeting vector pKU-V.

Plasmid pKU-V was transformed into *P. putida* KTU-TG using the electroporation method. Screening of the mutant strain was carried out as described above. The resulting mutant strain was designated as *P. putida* KTU-TGV.

*Construction of P. putida KTU-TGF and KTU-TGVF by chromosomal scarless modification.* The upstream and downstream fragments of flagellar operon were obtained by PCR with Phanta HS Super-Fidelity DNA Polymerase using primers F-1/F-2 and F-3/F-4, respectively. Subsequently, the two fragments were ligated together using overlap PCR. The generated fragment was digested with *Eco*RI and then subcloned into the same restriction site of pKU to create the gene knockout vector pKU-F.

Plasmid pKU-F was transformed into *P. putida* KTU-TG and KTU-TGV using the electroporation method. Screening of the mutant strains was carried out as described above. The resulting mutant strains were designated as *P. putida* KTU-TGF and KTU-TGVF.
